# Using transcripts to refine image based cell segmentation with FastReseg

**DOI:** 10.1038/s41598-025-08733-5

**Published:** 2025-08-20

**Authors:** Lidan Wu, Joseph M. Beechem, Patrick Danaher

**Affiliations:** Bruker Spatial Biology, Seattle, WA 98105 USA

**Keywords:** Bioinformatics, Quality control

## Abstract

Spatial transcriptomics (ST) faces persistent challenges in cell segmentation accuracy, which can bias biological interpretations in a spatial-dependent way. FastReseg introduces a novel algorithm that refines inaccuracies in existing image-based segmentations using transcriptomic data, without radically redefining cell boundaries. By combining image-based information with 3D transcriptomic precision, FastReseg enhances segmentation accuracy. Its key innovation, a transcript scoring system based on log-likelihood ratios, facilitates the quick identification and correction of spatial doublets caused by cell proximity or overlap in 2D. FastReseg reduces circularity in boundary derivation, and addresses computational challenges with a modular workflow designed for large datasets. The algorithm’s modularity allows for seamless optimization and integration of advancements in segmentation technology. FastReseg provides a scalable, efficient solution to improve the quality and interpretability of ST data, ensuring compatibility with evolving segmentation methods and enabling more accurate biological insights.

**Supplementary Information:**

The online version contains supplementary material available at 10.1038/s41598-025-08733-5.

## Introduction

Accurate cell segmentation is foundational in spatial transcriptomics, as it delineates individual cells and assigns transcript molecules to their cellular origins, influencing all subsequent analyses. Segmentation errors can contaminate single-cell expression profiles with neighboring cell transcripts, introducing spatially dependent bias that confounds analyses. For instance, differential expression analyses of a given cell type across spatial domains may be skewed by errors that arise from poorly segmented cells, overshadowing genuine biological signals^1^.

**Fig. 1:**
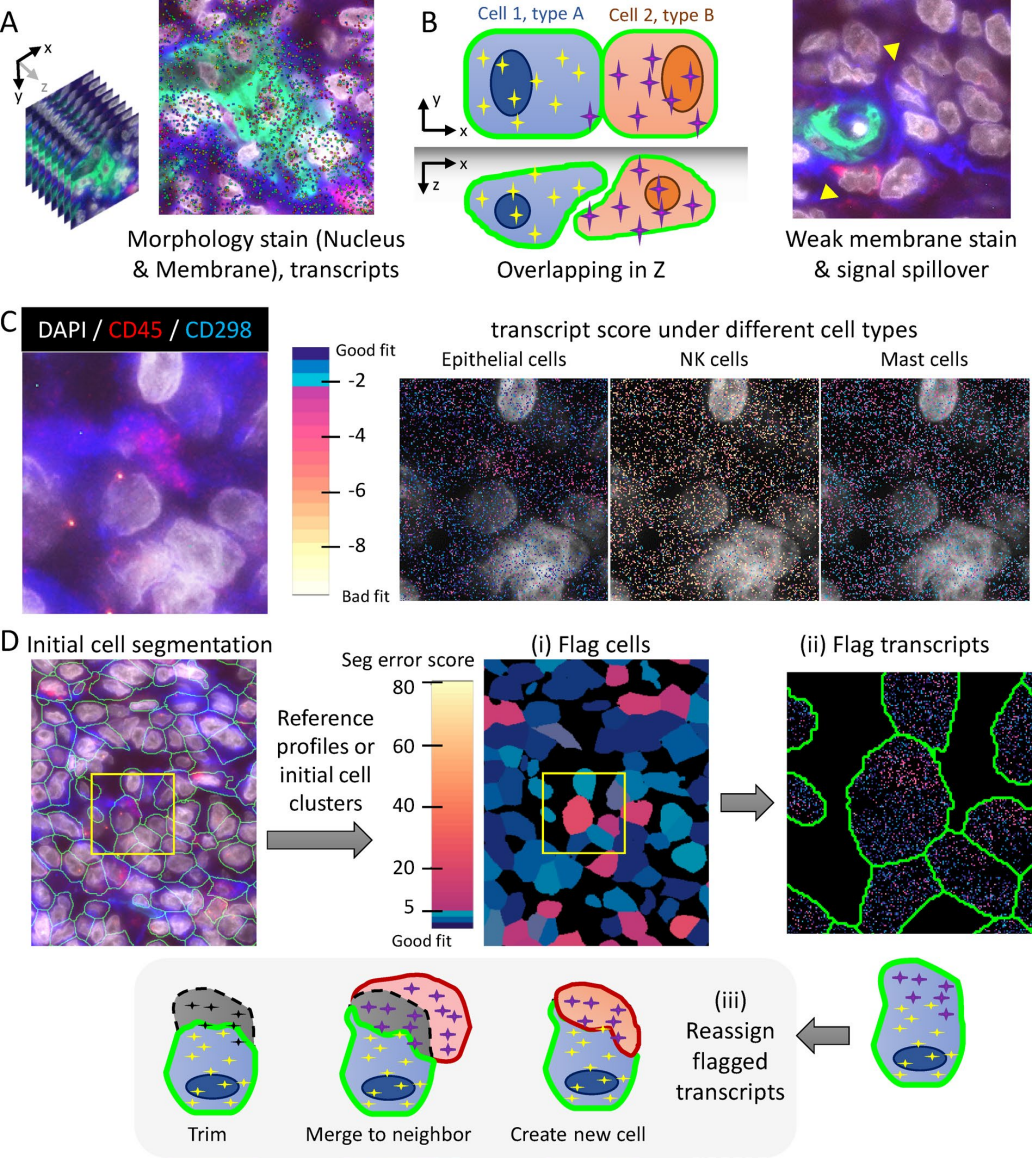
FastReseg, an algorithm for improved cell segmentation in spatial transcriptomics. (**A**) Visualization of typical spatial transcriptomics data showing morphology stains for nucleus and membrane alongside transcript distribution (dots colored by gene identities), providing context for segmentation. (**B**) Common challenges in image-based cell segmentation, including limited resolution to resolve overlapping cells in the z-axis, weak or incomplete membrane stain in real-world tissue samples (yellow arrows), and lateral spillage of optical signals in densely packed tissues. Example images in (**A**) & (**B**) are from a melanoma dataset, where morphology is visualized using antibodies against protein markers (CD298: Blue, PanCK: Green, CD45: Red) and nuclear stain (DAPI: gray). (**C**) Spatial pattern of transcriptional scores (right) based on each gene’s log-likelihood expression under reference cell types (Epithelial, NK, Mast cells) could help identify the presence of different cell types spatially, while validated by the orthogonal antibody staining (left, CD298: blue, CD45: red, DAPI: gray). Different color hues and intensities of the dots indicate the goodness-of-fit of each transcript (dot) under a given reference cell type as shown in the color bar, ranging from good (blue) to poor (red/yellow). See Methods section for calculation of tLLR scores plotted here. (**D**) Overview of the FastReseg workflow. Starting with initial cell segmentation and cluster-specific reference expression profiles, FastReseg scores each transcript based on its goodness-of-fit with respect to most probable cell type given the expression profiles of corresponding host cells and then flags cells with high spatial dependency in the spatial pattern of their transcript scores as having putative segmentation errors (i). Within the flagged cells, FastReseg can further identify the transcripts with poor fit, segregate them into spatially distinct groups and flag them as contaminating transcripts from different neighboring cells (ii). In the correction phase (iii), FastReseg evaluates each flagged transcript group’s expression and physical spatial context, deciding refinement actions such as trimming transcripts to extracellular space, merging with neighboring cells, or reassigning them to newly created cells based on a set of heuristic rules, which aims to resolve cell segmentation errors and improve transcript assignment accuracy. See Fig. 4 and Methods section for the detailed process.

Most spatial transcriptomics studies (Fig. 1A) employ image-based cell segmentation, which involves immunofluorescence (IF) staining of cellular morphological markers like the nucleus and membrane to identify cell boundaries. Classical methods like the watershed algorithm^2^ and nucleus expansion have been largely superseded by more advanced deep-learning techniques such as Cellpose^3^ and Mesmer^4^. However, limitations in image quality of real-world tissue samples (Fig. 1B) —such as weak staining, ambiguous boundaries, or poor 3D resolution—can lead to segmentation inaccuracies that propagate downstream.

Transcript-based methods offer an alternative by segmenting regions of distinct local expression profiles^5,6^. But they can face difficulties at the borders of closely related cell types or in low-transcript-density regions, limiting their usage in many cell-driven biological studies. Baysor^7^, JSTA^8^ and Proseg^9^ represent a new class of hybrid approach which combine transcript data with image priors. However, in practice the image priors are usually limited to the nuclear segmentation results, and the transcriptomic data frequently dominates the final segmentation outcome. This strong dependence on transcript data risks introducing circularity to analyses, with the gene expression patterns we wish to study influencing how the single cell expression matrix is defined in the first place. Moreover, the existing hybrid methods often require substantial computational resources^7–10^ due to the volume of transcript data and sophisticated modeling involved, and thus struggle to generate satisfactory cell-level segmentation for densely packed tissue samples.

To address these challenges, we introduce FastReseg, an R package designed to enhance the precision of cell segmentation by leveraging transcriptomic data to correct and refine initial image-based segmentation results. FastReseg processes spatial transcriptomic datasets, along with their initial cell assignment provided by image-based cell segmentation, through a three-tiered modular approach: it first assesses cells for potential segmentation inaccuracies, then identifies misassigned transcript groups within those likely erroneous cells, and finally reassigns these mislocated transcripts to their appropriate cellular origins. This stepwise progression allows users the flexibility to halt the process after any stage to examine intermediate results, adapting the workflow to suit specific research needs at various analytical depths. The modular structure also simplifies the parameter optimization for each module, facilitating the adaptation of this tool in diverse research contexts.

FastReseg’s benefits are multifold: it preserves the integrity of initial image-based segmentation, using transcript evidence for correction only where warranted. This strategy ensures that gene expression data used to study cell functions downstream is not directly compromised by the very methods intended to define cell boundaries. Besides, as image-based segmentation methods continue to evolve, FastReseg remains a relevant tool, complementing these advancements with its additional layer of transcript-based refinement. Moreover, FastReseg is designed to be fast and memory-efficient, handling computations on a cell-by-cell basis which allows saving the most expensive computations for the cells flagged as high likelihood of segmentation error. Recognizing the three-dimensional nature of tissue structures, FastReseg considers transcripts’ full 3D positions during modeling, making it particularly suited to modern, high-resolution spatial transcriptomics datasets.

Through FastReseg, we offer a novel and practical solution to one of the most pressing challenges in the field of spatial transcriptomics, propose a framework that could significantly improve the accuracy and reliability of cell segmentation and thereby enhance the overall quality of biological insights derived from spatial data.

## Results

### Concept of transcript scoring

FastReseg works on the premise that each RNA transcript offers evidence supporting or refuting the presence of a particular cell type at its location. For example, a CD68 transcript suggests the presence of a macrophage, while simultaneously arguing against the cell being a T cell. This foundational idea is encapsulated by deriving a log-likelihood ratio ($\:tLLR$) for each gene under each expected cell type against the most probable cell type of given gene across the reference expression profiles for the dataset of interest. These log-likelihood ratios, which we term tLLR scores, are used to score every transcript in every cell and serve as the foundation of FastReseg’s operations. While one could obtain reference profiles from either external single-cell RNA sequencing (scRNA-seq) dataset or query data itself (see Methods section), the primary requirement is that they accurately represent the expressional centroids of the major cell types present in the query dataset. Capturing fine cell types, on the other hand, is less critical for FastReseg, as the differentiation between closely related subtypes often offers limited resolution in transcript-based local evidence and thus is of minimal utility in detecting and correcting segmentation errors.

These “transcript scores” form the core operating mechanism of FastReseg and quantify how well each transcript’s expression profile matches the expected profile of its designated cell type. This involves assessing the consistency of transcripts within a neighborhood defined by initial image-based cell segmentation—ensuring they align in terms of log-likelihood ratios against the same cell type—and examining their spatial distribution. Such scores not only represent the goodness-of-fit of the transcript to the cell type but also highlight any spatially dependent patterns that may indicate the presence of contaminating source cell and thus segmentation inaccuracies (Fig. 1C). This dual approach ensures that both expression and physical space are considered, enhancing the accuracy of detecting and correcting segmentation errors.

### FastReseg framework

**Fig. 2:**
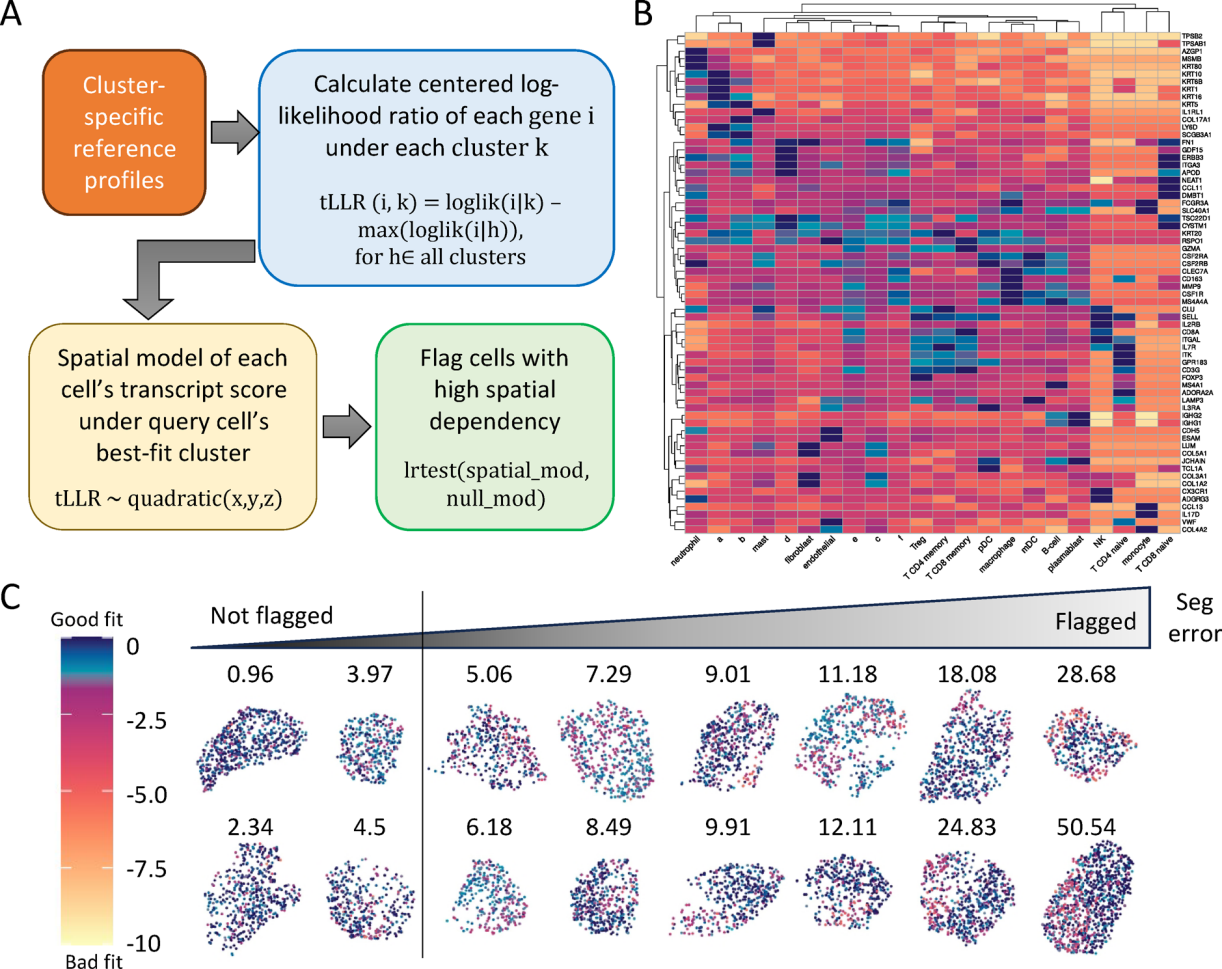
Detection of cells with putative segmentation errors based on local transcriptional profiles in both expression and physical space. (**A**) Flowchart of how FastReseg identifies cells with potential segmentation errors. Cluster-specific reference profiles are utilized to calculate the centered log-likelihood ratio (tLLR score) for each gene under each reference cell cluster/type, where tLLR (i, k) is derived by subtracting the maximum log likelihood of gene i across all clusters from its log likelihood under cluster k. A linear regression model is then trained to simulate the spatial pattern of each cell’s transcriptional score under its best-fit cluster given the overall expression profiles of query cell observed in current cell borders. FastReseg would further evaluate the degree of spatial dependency of the transcript score pattern of each query cell via log-likelihood ratio test between the spatial-dependent model and the invariant null model. Cells with high spatial dependency are flagged as cells with putative segmentation errors. (**B**) Heatmap of transcript tLLR score for top marker genes (rows) across different reference cell types (columns). The reference cluster-specific profiles (see “example_refProfiles” object inside the package) were derived from an example spatial transcriptomics dataset for melanoma tissue section whose cell types were assigned based on semi-supervised cell typing algorithm with novel clusters using “a” to “f” letters as names. The color scale, matching (**C**), illustrates the range from good to bad fit, highlighting the variability of score for classic marker genes in different reference cell types. (**C**) XY scatter plots of transcript tLLR scores within example cells with varying likelihood of segmentation error, as determined by the degree of spatial dependency (defined as –log_10_(p), and annotated on top of each cell) of their tLLR patterns under their most probable cell types. Cells with spatial dependency scores higher than the provided cutoff (default to 5, shown as the vertical line above) are flagged as improperly segmented cells.

**Fig. 3:**
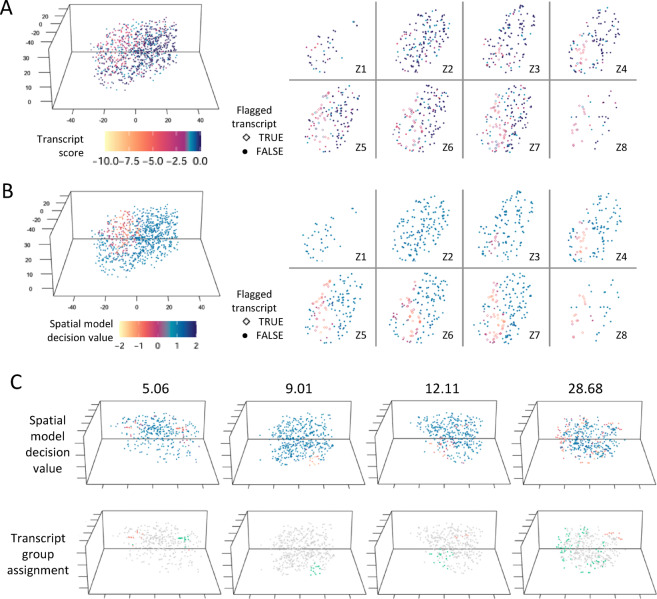
Detection and segregation of misassigned transcript groups. (**A**) XYZ scatter plots of transcript tLLR scores for a flagged example cell with spatial dependency score of 50.54, visualized both in 3D (left) and across multiple 2D Z-slices (right). Each point represents a transcript, colored by transcriptional score under the most probable cell type given the cell’s overall gene expression profile. This visualization highlights the spatial distribution of potentially misassigned transcripts within the cell. (**B**) XYZ scatter plots of the decision values produced by a Support Vector Machine (SVM) model trained to predict whether a given transcript would have transcript score below the − 2 cutoff given its spatial coordinate within the same cell shown in (A). Negative decision values correspond to below-cutoff poor-fit prediction by the SVM model. In both (**A**) and (**B**), the shape of the points corresponds to the classification predicted by the SVM-based spatial modeling of the given cell. Transcripts predicted to have transcript score below cutoff are treated as flagged misassigned transcripts and depicted as hollow diamond points in the XY scatter plots for multiple Z-slices on the right of (**A**) and (**B**). The difference in the spatial pattern of transcript tLLR scores and SVM decision values of same cell provides insights into the spatial constraints on predicting misassigned transcripts. (**C**) Additional examples of cells with varying degrees of spatial dependency on their transcript scores (5.06, 9.01, 12.11, 28.68). For each cell, transcripts predicted as poor-fit with negative decision values by the SVM are further segregated into spatially distinct groups, shown in different colors at bottom panel. These groups indicate the likely origins of those flagged misassigned transcripts from different neighboring source cells, providing a basis for further targeted reassignment and refinement of cell segmentation.

**Fig. 4:**
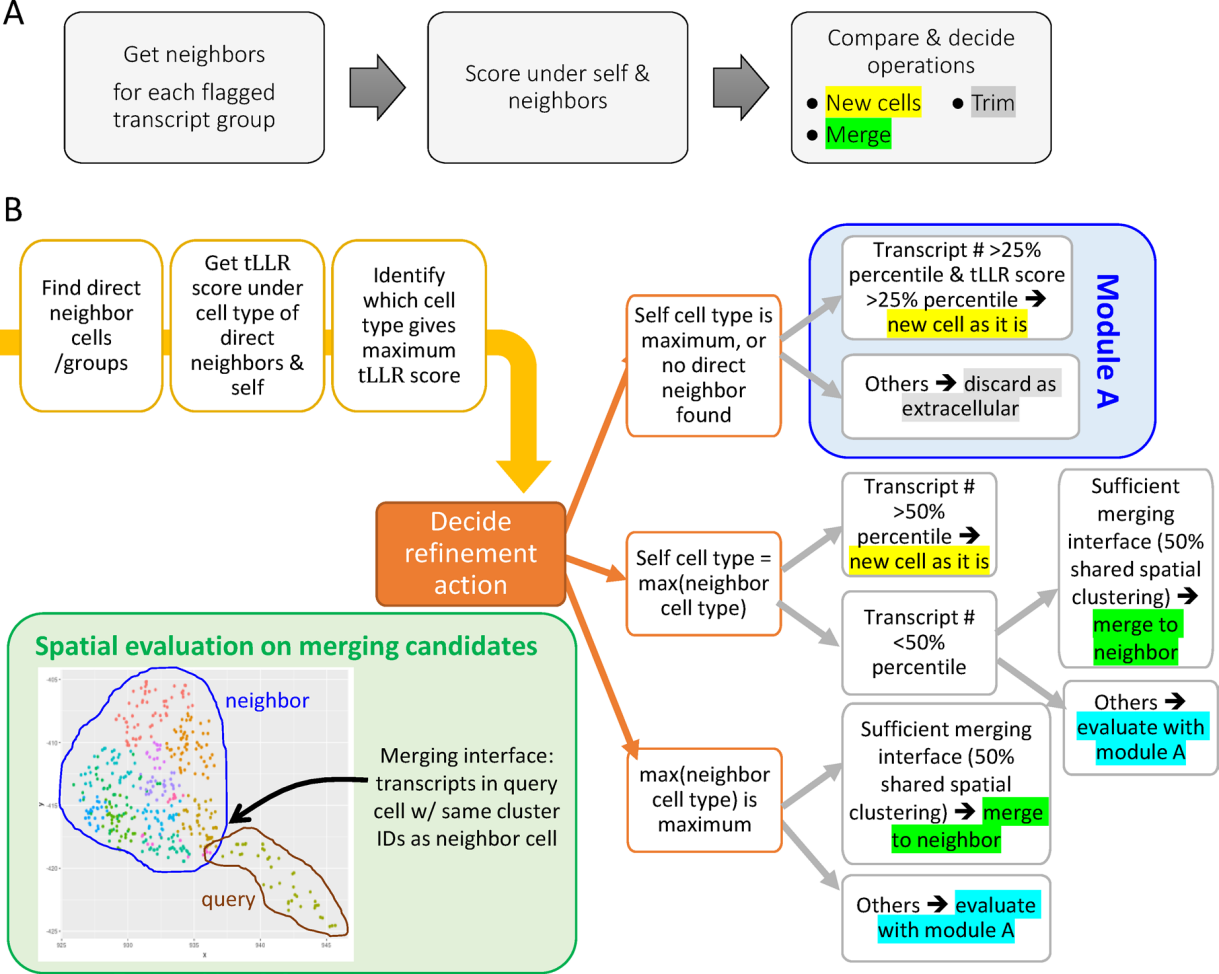
Heuristic-based approach to evaluate and reassign poorly fitted transcript groups. (**A**) High-level workflow for transcript reassignment, involving the identification of direct neighboring cells or groups in space, assessing total transcript tLLR scores under the most probable cell types of both the cell/group itself and its neighbors, and determining refinement actions such as creating new cells, trimming, or merging. (**B**) Decision tree and detailed criteria for deciding refinement actions based on the analysis of physical and expression contexts with respect to baseline distribution of transcript number and total transcript score of relevant reference cell types observed in the query dataset under the original cell segmentations. The inset plot at lower left corner in (**B**) shows the XY scatter plot of transcripts within an example pair of merging candidates, where Leiden clustering is performed on transcripts’ spatial coordinates to segregate them into different spatial clusters shown in different colors. The merging interface, as defined by the fraction of query transcripts sharing the same spatial clusters as the transcripts in its merging candidate partner, is used to evaluate whether sufficient physical contact exists between the pair of merging candidates, emphasizing the necessity of spatial constraint on a valid merging event.

FastReseg’s workflow (Fig. 1D) is a three-tiered process which begins with two essential inputs: the initial results of cell segmentation, typically derived from morphology images with transcript-level cell assignment, and a matrix detailing transcript scores across all genes and cell types (Fig. 2A-B). The first tier of the process evaluates each cell’s transcriptional spatial pattern with a “spatial doublet test”, which analyzes transcript scores under each cell’s most probable cell type given its overall single-cell expression profiles to detect spatial dependencies. Cells enriched with poorly fitting transcripts in localized areas are flagged for potential segmentation errors (Fig. 2C). This test, which can be performed very rapidly and massively in parallel, lets us redact cells with no evidence of segmentation error from more computationally intensive downstream operations. The second tier of the process focuses on cells flagged from the spatial doublet test and attempts to segregate high-score transcripts (well-aligned with the cell type) from low-score transcripts (poor fits) via a spatial model trained for each flagged cell (Fig. 3). Regions enriched in low-score transcripts are considered candidates for contaminating transcripts due to segmentation errors. Depending on analytical needs, these erroneous regions can either be excised to clean up the dataset or subjected to further refinement in third tier. In the final tier, FastReseg applies a series of heuristics to determine the corrective actions (Fig. 4) for cells with identified misassigned transcript groups: trimming these areas from the dataset, merging them into an existing neighboring cell, or establishing them as new, independent cells. These decisions are guided by the spatial and expression context of the transcript groups to ensure corrections respect both the physical and expressional landscapes of the tissue. By integrating sophisticated transcript scoring with practical segmentation refinement in a modular pipeline, FastReseg offers a powerful tool for researchers seeking to delve deeper into the cellular complexities of biological tissues.

### Spatial doublet test

FastReseg’s “spatial doublet test” is designed to identify cells whose expression profiles might erroneously include transcripts from adjacent cells, a situation we term “spatial doublets”. This concept extends traditional doublet detection from scRNA-seq by incorporating spatial context. Unlike scRNA-seq doublets which merge entire cellular transcripts, spatial doublets often involve direction-dependent contamination from fragments of adjacent cells. The precise transcript locations in spatial transcriptomics enables detection of such asymmetric patterns of inconsistency, going beyond the capabilities of traditional “bag-of-RNA” approach.

**Fig. 5:**
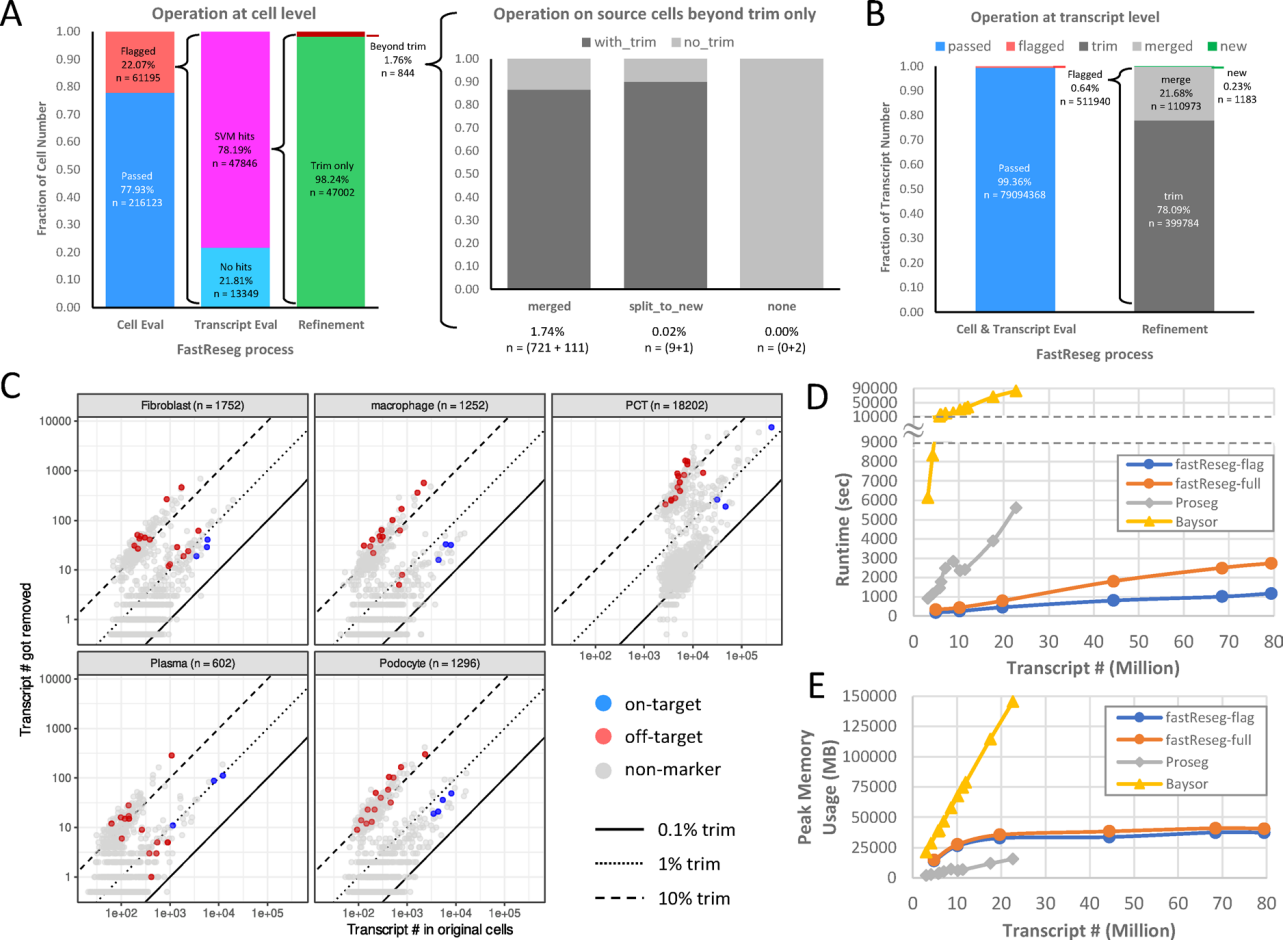
Performance evaluation of FastReseg on an example kidney dataset. (**A**) Bar plots on the composition of actions taken at cell level throughout the FastReseg workflow. While majority of cells (98.24%) with misassigned transcript groups identified by SVM modeling have received trimming during the transcript refinement stage, the bar plot at the center highlights the different refinement actions applied to the original host cells on top of trimming some transcripts to extracellular space, including merging those misassigned transcripts to neighboring cells (“merged”), designating them as new cells (“split_to_new”), or returning back to the host cell (“none”, *n* = 2 cells). Color legend of “with_trim” and “no_trim” indicates whether the host cells received more than one refinement actions. (**B**) Bar plots on the breakdown of operations conducted at the transcript level, illustrating the extent of transcript reassignment across the dataset. (**C**) Scatter plots of transcript number of each gene for each cell type. Only cells that received transcript trimming are included in this before-vs-removed comparison to show the impact of FastReseg refinement on original host cells under different cell types. The cell number included in each subplot is noted in the subtitle next to the cell type of interest. Dots for each gene are colored based on whether they are on-target or off-target marker genes for the cell type of interest. Genes that are not considered as mutually exclusive markers are colored in gray as “non-marker”. See Methods section (Table 1) for the mutually exclusive marker genes used in this analysis. The solid, dotted and dashed lines indicate trimming at different percentages (0.1%, 1%, 10%). (**D**) Runtime and (**E**) peak memory usage of two FastReseg pipelines (flag-only and full-pipeline) along with other transcript-based cell segmentation methods (Proseg and Baysor) on datasets containing different transcript numbers. The statistics reported are based on processing using 75% cores of an Amazon r5b.4xlarge instance (16 vCPU, 128GB memory) unless otherwise noted in Methods section. The input spatial datasets were subsets of the CosMx kidney dataset either with different numbers of fields of view (Table 2) or from different tissue sections (Table 3), each containing varying cell numbers in their original cell segmentation.

Among various techniques appropriate for detecting spatial doublets, FastReseg uses polynomial regression to efficiently tackle this issue, making the assessment of millions of cells amenable. Specifically, FastReseg employs a quadratic model that predicts transcript scores based on the three-dimensional positions of transcripts within a cell (Fig. 2A). For each cell, the algorithm compares the fit of this quadratic model to a null model, where the position is assumed not to influence transcript score. The p-values of likelihood ratio test between these two models serve as a metric to evaluate the presence of spatial doublets. Cells exhibiting small p-value suggest significant spatial dependencies in their transcript distributions and are flagged as potentially suffering from segmentation errors (Fig. 2C). This approach not only highlights the cells with likely errors but also effectively filters out those with no apparent issues. With a reasonable cutoff ($\:p\:\le\:\:{10}^{-3}\:or\:{10}^{-5}$), FastReseg excludes 80-90% of cells with no strong evidence of segmentation errors from further analysis (Fig. 5A), substantially reducing unnecessary computational workload in subsequent steps of the pipeline.

### Detecting misassigned transcripts via support vector machine

For cells flagged by the spatial doublet test, the next crucial step involves identifying and isolating misassigned transcripts within these flagged cells. Given the fact that transcripts belong to the same source cell would stay in proximity to each other in physical space, FastReseg looks for spatially contiguous regions enriched with molecules of low transcript scores and considers transcripts residing in those regions to be the contaminants due to segmentation error irrespective to their individual transcript scores. Frameworks used in earlier works, such as hidden Markov random fields or building of molecular nearest-neighbor networks, are theoretically appealing but computationally expensive. FastReseg utilizes a more efficient strategy: a support vector machine (SVM)^11^ with a radial kernel is fitted to the spatial distribution of the assigned transcript scores within each flagged cell under each cell’s most probable cell type. The trained SVM model can effectively predict low versus high transcript scores based on their 3D positions within the query cell (Fig. 3A-B). The radial kernel is particularly suited for this task as it accommodates non-linear boundaries shaped by the physical contours of cells and can identify multiple, spatially distinct groups of low-score regions (Fig. 3C) that may emanate from different source cells in the neighborhood of the query cell.

The threshold defining low vs. high transcript scores is a critical turning parameter of the SVM-mediated detection of misassigned transcripts. FastReseg employs a default threshold of -2, which corresponds to a p-value of ~ 0.046 under the interpretation of transcript score as a log-likelihood ratio between two competing hypotheses about its cell type origin (see Supplementary Information). Adjusting this threshold higher can lead to a more aggressive approach which flags more molecules with intermediate evidence of poor fitting. Moreover, FastReseg offers the flexibility to modify SVM parameters, such as the gamma and cost, for fine control on the responsiveness of misassignment detection with respect to singlet transcript molecules. While the default value of SVM parameters set by the FastReseg package offers a commendable baseline for analyzing any new spatial dataset, users are free to explore and tailor their setup to the characteristics of the dataset of interest, such as the data richness—in terms of physical molecular density and gene content diversity—and the anticipated cellular morphology, whether it be round or exhibiting elongated protrusions. The impact of these parameter adjustments on FastReseg’s performance is illustrated in Supplementary Fig. S1, which serves as a guideline for parameter optimization.

### Heuristic-based reassignment of flagged transcripts

After isolating misassigned transcripts and segregating them into spatially distinct groups, FastReseg can proceed to reassign those flagged transcript groups to their appropriate source cells. We opted to employ heuristic rules rather than more computationally intensive, algorithmically complex models such as the Markov Random Fields (MRFs) employed by Baysor^7^, or the training-required tandem deep neural networks (DNNs) utilized by JSTA^8^, or the Monte Carlo based inference approach adopted by Proseg^9^. Although these sophisticated models are theoretically appealing, they often do not offer proportional benefits in the context of sparse or fragmented datasets and might give spurious results disagreeing with morphological stain because of insufficient data points. Instead, the heuristic approach is chosen to strike an optimal balance between computational efficiency and the accuracy of segmentation refinement in spatial transcriptomics datasets which frequently comprise thin tissue sections harboring many partial cells. These partial cells typically contain limited transcriptional information, reducing the utility of applying complex models. The heuristic rules guiding FastReseg’s refinement process are grounded in a mechanistic understanding of cellular biology and the realistic distribution of transcripts within and across cells (see Methods section). As depicted in Fig. 4, these rules pragmatically evaluate factors such as the distribution of transcript number and score for each cell type within the query dataset, the consistency of transcript profiles post-merging, the extent of spatial connection between merging candidates, and the feasibility of trimming transcripts to extracellular spaces when evidence suggests no presence of valid new-cell or merging event. In the next section, we will discuss the segmentation refinement outcomes generated with real-world spatial transcriptomics dataset.

### Evaluation of FastReseg performance

We evaluated FastReseg using a spatial transcriptomics dataset published by previous study^12^. This dataset was generated from archived clinical biopsies of human kidney samples at various stages of lupus nephritis and was collected using NanoString CosMx^®^ platform and a 960-plex RNA panel. The cell segmentation coming with this dataset was performed using pretrained Cellpose models on 2D projected IF images of several morphological markers (DAPI, CD45, PanCK, CD20 and CD298). Thanks to the advanced machine-learning cell segmentation models, cell borders defined in the original cell segmentation results show strong agreement with the rich information encoded in the morphological images but fail to capture the 3D volumes of cells properly and suffer from border error in regions with weak or incomplete staining, an inevitable issue observed in real-world tissue samples. With these segmentation results, the original study has performed supervised cell typing against a reference expression matrix constructed based on external scRNA-seq datasets from Human Cell Atlas and other publications. While different cell clustering approaches could be used without significant impact, we leveraged the existing cell typing results for simplicity to generate the cluster-mean profiles observed in this CosMx kidney dataset and use them as reference profiles to derive transcript score for each gene under each expected cell type.

In Fig. 5A-B, we quantify the FastReseg outcomes across its modular process at both transcript and cell level when processing the entire kidney dataset through full pipeline. In first-tier process, FastReseg’s spatial doublet test flagged 22.07% of cells from the original segmentation results due to significant spatial dependency (p$\:\:\le\:\:0.001$) in their transcript scores patterns. In second-tier process, 78.19% of those flagged cells were further identified by SVM spatial modeling to contain localized zones enriched of low-score transcripts (“SVM hits”). These zones, containing 0.64% of total intracellular transcripts, underwent further evaluation in the third-tier process of segmentation refinement. In this final tier, the heuristic-driven decision-making process led to over 98% of cells with SVM hits having their misassigned transcripts removed to extracellular space, impacting 78.09% of flagged transcripts. Additionally, 21.68% of all misassigned molecules were merged into neighboring cells that showed consistent transcriptional profiles in expression domain, affecting 1.74% of flagged cells. Notably, 10 original cells with SVM hits gave rise to new cells, representing 0.23% of identified misassigned transcripts. A very small subset of flagged cells (*n* = 2), despite showing SVM hits, received no refinement action; this typically occurred when the particular group of identified misassigned transcripts was best matched with its original host cell, possibly due to only marginal spatial dependency from only a handful of source molecules. This nuance emphasizes the stringency and robustness of transcript reassignment within FastReseg. Noticeably, some host cells in original segmentation could receive multiple types of refinement action (Fig. 5A, center panel), illustrating the diverse outcomes possible with FastReseg’s refinement process. For detailed examples of the refinement actions undertaken by FastReseg across various stages of the full pipeline, refer to Supplementary Fig. S2.

While it’s challenging to establish ground-truth segmentation for large spatial transcriptomics dataset, we sought to track the transcript reassignment for major cell types via a list of canonical mutually exclusive markers (Table 1). Figure 5C compares the number of transcripts originally observed within cell borders to the ones removed by FastReseg for different subpopulations of host cells that have received transcript reassignment. The on-target marker genes for major cell types showed a minimal trimming rate around 1%, confirming their relevance to the cells of interest. Conversely, transcripts from off-target marker genes, which are unlikely to be present in the host cells, were removed at higher rate, often exceeding 10%. The remaining genes (“non-marker”) exhibited a binary distribution in their trimming rate, as genes with high likelihood to be expressed within the cell type of interest tend to have low trimming rate within same bin as that of the canonical on-target markers. This selective behavior demonstrates the integrity of the FastReseg’s choice of transcript reassignment and helps to reduce the ambiguity of the resulting single-cell expression profiles, offering a clearer insight into the cellular biology within the sampled tissue.

### Processing efficiency and scalability

The whole CosMx kidney dataset comprises over 79 million transcripts across 123 fields of view (FOVs) each covering a 0.985 mm x 0.657 mm area with 0.4 ~ 1.2 million transcripts. All transcripts for each FOV are consolidated into one single file. The size of this large dataset poses a significant challenge for existing transcript-dominant cell segmentation methods, whose runtime and peak memory usage typically scale linearly with transcript quantity. For context (Fig. 5D-E; Table 3), we have observed 93 min runtime and 15.17 GB peak memory consumption for Proseg processing on a subset of the CosMx kidney dataset containing 19 million transcripts, while Baysor took > 23 h and 142 GB peak memory on the same data subset.

In contrast, FastReseg is designed with maximizing computational efficiency in mind and engineered to handle big datasets that are often required for studying complex biological problems. FastReseg’s workflow processes transcriptional data on a per-input-file basis, markedly reducing the memory footprint required for large dataset and facilitating effective parallelization across multiple CPU cores. On an Amazon r5b.4xlarge instance (16 vCPUs, 128GB total memory) using 75% available cores (i.e. 12 cores), FastReseg maintained a stable peak memory usage at 32 ~ 37 GB across input size from 20 ~ 79 million transcripts during flag-only processing that evaluates and separates misassigned transcripts (Fig. 5E). The full pipeline, which adds more computationally demanding steps of segmentation refinement, used just 2 ~ 5 GB more peak memory on the same input. This relatively constant peak memory usage was observed when the number of per-FOV input files exceeded the number of usable CPU cores, and originated from FastReseg’s per-file data ingestion and its architecture of parallelized computation. When input files are fewer than the usable cores (e.g. the first 2 data subsets with 5 or 10 FOVs, Table 2), FastReseg’s peak memory usage scales linearly with the transcript number. If reducing peak memory consumption is a priority, further optimizations can be achieved by splitting transcripts into smaller image tiles with fewer data per input file or adjusting the percentage of cores utilized by FastReseg via its `percentCores` argument.

FastReseg’s modular, tiered design targets intensive computation only to the cells that mostly need segmentation correction, resulting in similar runtimes between flag-only and full pipeline with only 2 ~ 26 min difference depending on the number of transcripts involved. When processing a typical benchmarking load of 4.8 million transcripts, FastReseg completed the flagging in just 3.11 min and the full pipeline in 5.76 min, making it ~ 24x faster than Baysor (138.8 min) and 3x faster than Proseg (19.28 min). For the full 79 million transcript dataset, which are unmanageable by both Baysor and Proseg due to their prohibitive peak memory consumption, FastReseg adeptly completed the full pipeline within 45.5 min, maintaining a peak memory usage of only 45 GB. This blend of speed and efficiency makes FastReseg a feasible tool for processing large-scale spatial transcriptomics datasets, enabling complex biological research on big sample collections to proceed smoothly.

### Comparison with transcript-dominant approaches

Transcript-based segmentation methods differ fundamentally in how they incorporate prior cell boundaries. FastReseg follows a “revision-based” strategy which selectively adjusts cell boundaries only when supported by local transcript evidence. In contrast, Proseg and Baysor adopt more aggressive “reassignment-based” approaches where transcript assignment is primarily driven by local pattern, with penalties applied based on distance from the initial cell centroids.

**Fig. 6:**
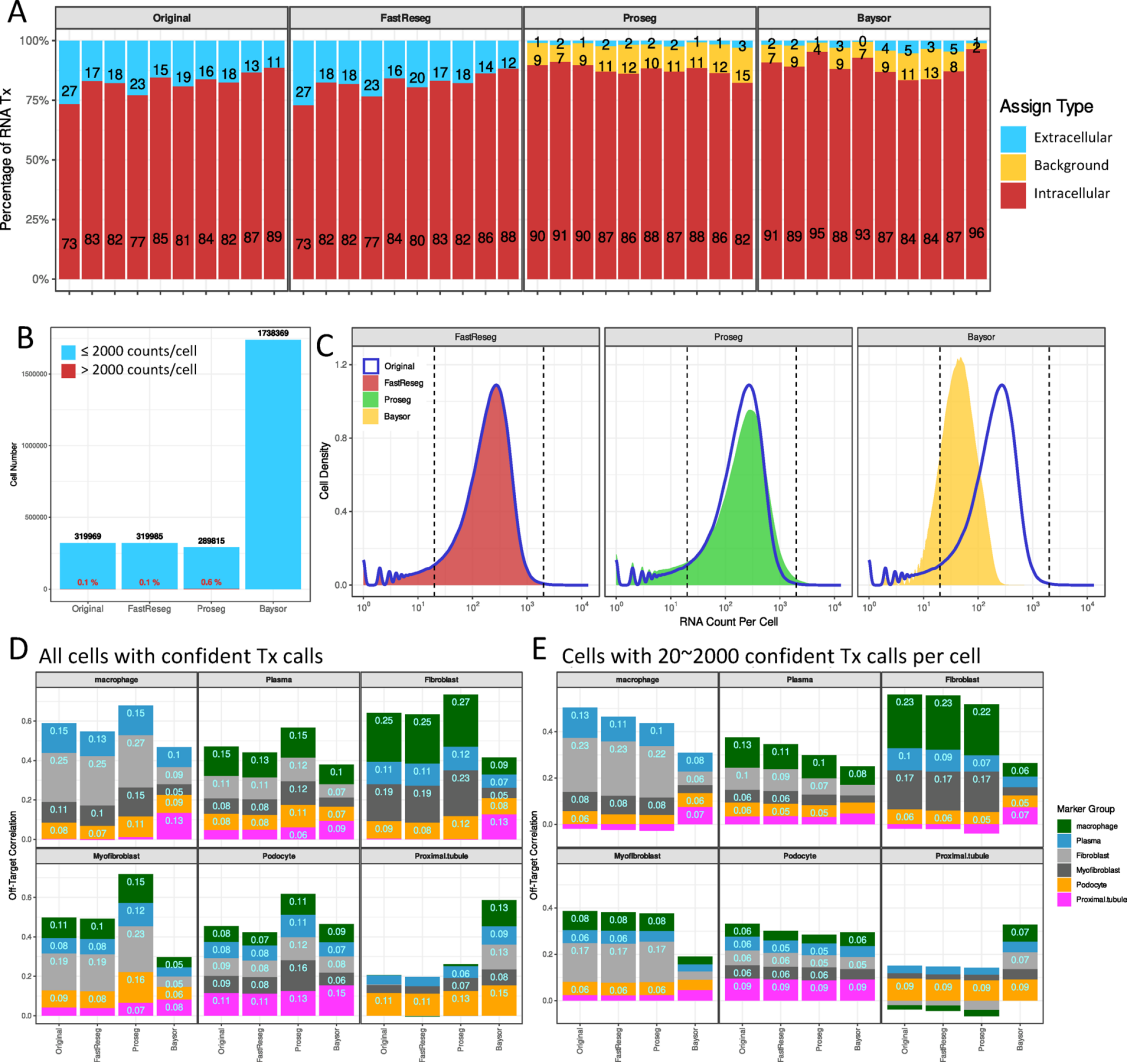
Comparison of segmentation outcomes across different segmentation methods. (**A**) RNA transcript assignment composition across 10 tissue samples from the CosMx kidney dataset using different segmentation methods: original image-based segmentation, FastReseg full-pipeline, Proseg and Baysor. All transcript-based segmentation methods were provided with the image-based segmentation outcomes as prior. Transcripts labeled as “background” include those marked as “background” by Proseg or with reported assignment confidence < 0.9 by Baysor. (**B**) Bar plots showing the number of RNA-containing cells identified by different methods on the full CosMx kidney dataset. Red labels indicate the percentage of cells containing over 2000 RNA transcript counts. (**C**) Histogram of RNA count per cell across methods. The original image-based segmentation is shown as a blue line, while transcript-based methods are displayed as filled curves in separate subplots. Vertical dashed lines mark threshold at 20 and 2000 RNA counts per cell. (**D**,** E**) Per-sample average off-target correlation between marker groups (Table 1), computed from single-cell expression profiles of all 10 individual CosMx kidney samples (Table 3). Correlation was measured using the Pearson correlation of single-cell metagene scores between mutually exclusive marker groups. (**D**) includes all cells with at least one RNA transcript, while (**E**) includes only cells with 20 ~ 2000 RNA transcripts. Each subplot shows a query marker group’s correlation with other groups as stacked bar plots, with values > 0.05 labeled. Each bar along the x-axis corresponds to one segmentation method, while each color corresponds to one marker group. The color legend for marker groups is shared between (**D**) and (**E**).

As shown in Fig. 6A, both Proseg and Baysor assign significantly more transcripts as intracellular than the original segmentation. Much of this increase arises from regions that have spatially diffusive transcript patterns but lack clear morphological evidence of cell presence. At the single-cell level (Fig. 6B), Proseg recovers fewer cells but generates a small population (~ 0.6%) of extreme outliers with > 2000 RNA transcripts per cell and 2 ~ 3.6x larger volume. These outliers occur near tissue edges, where image-based segmentation leaves large cell-free regions nearby. Besides, Proseg results also exhibit a systematic rightward skew in the RNA count distribution (Fig. 6C) with more cells containing elevated transcript levels. Baysor, by contrast, over-segmented cells, resulting in ~ 5.5x more cells, each with fewer transcripts due to its emphasis on expression purity. These characteristics behaviors are further illustrated in Supplementary Fig. S3, which provides visual examples of segmentation outcomes.

These segmentation behaviors directly influence downstream single-cell expression profiles. When analyzing off-target correlation across all cells (Fig. 6D), Proseg exhibits inflated correlation values driven by its high-count outlier cells. But when filtering for cells with 20 ~ 2000 transcripts (Fig. 6E), all transcript-based methods reduce off-target correlation from the original values, with Baysor showing the largest drop owing to its smaller, lower-count cells. FastReseg, meanwhile, preserves the original transcript count distribution while offering a modest yet consistent reduction in off-target correlation. Crucially, Fig. 5C reveals that FastReseg’s conservative approach still achieves meaning biological refinement as it trims off-target markers far more than on-target ones, enhancing the interpretability and purity of expression profiles without overhauling the segmentation landscape.

## Discussion

We introduce a novel category of cell segmentation algorithm with FastReseg, which employs transcriptomic data to rectify apparent errors in prior image-based segmentation, rather than defining cell segmentation boundaries dominantly from transcriptional data. This methodology distinguishes itself from other transcript-based segmentation algorithms by reducing the risk of analytical circularity and enhancing computational efficiency. FastReseg’s adept integration of 3D cellular structures through transcriptional data into its analysis framework enables it to pinpoint segmentation inaccuracies that evade detection in conventional 2D imaging approaches. Since it functions to evaluate and refine the output of existing cell segmentation, FastReseg will remain relevant and valuable even as image-based segmentation techniques continue to advance.

The workflow of FastReseg consists of four distinct steps: scoring transcripts with respect to their initial host cells, identifying cells with putative segmentation errors through the “spatial doublet” test, flagging misassigned transcripts, and decisively managing the fate of these transcripts. We present this methodology as a versatile and easily extensible framework, with FastReseg being its first instantiation. This modular design allows independent enhancement of each component and easy integration of future improvements. Additionally, FastReseg’s architecture excels in processing large datasets, a common challenge in transcript-based segmentation. Its ability to manage extensive data efficiently positions it as a practical solution for large-scale spatial transcriptomics studies.

FastReseg’s core methodology involves scoring each transcript for its fit within its respective cell, effectively transforming the discrete gene identities into interpretable likelihood ratios. This simplification facilitates broader applicability across diverse gene panels and enables rapid detection of segmentation errors with minimal overhead when coupled with the spatial doublet test. We anticipate this scoring system to be a valuable tool for other applications in transcript-driven segmentation analysis.

FastReseg employs a conservative strategy for flagging potentially misassigned transcripts, requiring both poor transcriptomic fit based on gene identity, and spatial inconsistency with the shape of the host cell. This dual constraint minimizes the risk of circularity—where the same transcript data can unduly influence both the correction and resulting expression profiles. The current SVM model within FastReseg effectively identifies the most apparent errors, thereby improving the accuracy of single-cell expression profiles. Users can adjust parameters such as gamma and cost to fine-tune the model’s sensitivity to spatial versus expression-based discrepancies, allowing adaptation to different tissue types or data quality. While effective for clear-cut cases, further improvements could target subtle misassignments.

In datasets derived from thin tissue sections, partial cells are common. Removing all flagged transcripts based on FastReseg’s criteria typically results in cleaner and more reliable data for downstream analyses, while reducing the needs for extensive computation to segment cells with sparse transcriptional content. For more complex refinement scenarios, FastReseg applies a heuristic strategy to rapidly reassign flagged transcripts. This approach assumes that the initial image-based segmentation provides a high-confidence identification of cells, despite imprecision in exact boundary location or occasional errors caused by poor staining or limited 3D resolution. While FastReseg can generate new cells from localized misassigned transcript clusters within existing segmentation, it does not currently detect cells composed entirely of extracellular transcripts unassigned in the original segmentation. This reflects FastReseg’s design as a refinement tool that operates on the results of image-based segmentation. As a result, cells that are severely fragmented across multiple neighbors without a sufficient anchor transcript group may be missed. However, such cases are unlikely to yield reliable single-cell profiles due to low transcript abundance and ambiguity in morphological images. FastReseg’s conservative design ensures that these ambiguous transcripts are removed rather than misassigned, helping preserve the integrity of downstream analysis. If the foundational image-based segmentation is severely flawed, its error-detection features can still be used to evaluate segmentation quality; however, re-running image-based cell segmentation using modern machine learning techniques^13^ is recommended prior to apply FastReseg’s refinement process.

Because FastReseg’s workflow focuses its segmentation refinement actions exclusively on cells identified as spatial doublets, it currently does not target minor boundary inaccuracies that affect just one or two transcripts. Such small discrepancies typically have limited downstream impact but may introduce subtle artifacts in differential expression analysis on narrowly defined subpopulations. Enhancing FastReseg to support finer-grained boundary refinement is a valuable direction for future work.

In summary, FastReseg marks a pivotal development in the field of spatial transcriptomics, offering key innovations and advantages for the detection and correction of cell segmentation errors. Its powers in processing massive datasets efficiently, along with its modular and extensible framework, positions FastReseg as a vital tool for future research. As image-based segmentation technologies advance, FastReseg’s ability to refine segmentation outputs through the integration of transcriptional data ensures that it will continue to provide valuable insights and improvements in cell segmentation accuracy. Moving forward, the continuous enhancement of FastReseg’s capabilities, particularly in addressing cells in initial extracellular space and minor segmentation errors, will further cement its role as a crucial asset for researchers exploring the intricate landscape of spatial transcriptomics.

## Methods

### Reference transcript profile generation

The cluster-specific reference expression profiles represent the expected expression patterns of genes for distinct cell types within the sample and could be generated either from external sources or derived from the query dataset of interest. To get reference profiles from external sources, one can use previous single-cell RNA sequencing (scRNA-seq) or spatial transcriptomics datasets of similar tissue type and disease condition. Public databases for various cell atlas programs could be a good resource for such datasets. With the external dataset of choice, one can then calculate the average expression of each cell type in that dataset to be used as reference profiles. Alternatively, one could do cell typing on query dataset based on existing initial cell segmentation using any sensible clustering strategy, such as unsupervised Leiden clustering, reference-based supervised cell typing, or embedding-based label transfer. The initial cell segmentation, which could be based on the 2D morphological images of given query dataset, often has errors on the exact cell borders but is typically sufficient to capture the general gene expression differences across major cell types present in the sample. FastReseg package could then take the cell clusters assigned to query dataset and calculate the cluster-mean profiles for all provided clusters and use that as reference profiles.

### Scoring transcripts within cells

Using the reference profiles provided, FastReseg assigns every transcript an expression-based goodness-of-fit score, $\:tLLR$ (for “transcript log likelihood ratio”). The scoring process starts by scoring each gene under each cell type. FastReseg calculates the likelihood of gene $\:j$ given cell type $\:k$ based on the provided cluster-mean reference profiles $\:{\mu\:}_{k,\:j}$:

$$\:{\mu\:}_{k,\:j}={X}_{k,\:j}/\sum\:_{i\:\in\:all\:genes}{X}_{k,\:i}\:$$

where $\:{X}_{k,\:i}$ is the count of gene $\:i$ under cell type $\:k$ in cluster-mean reference profiles.

From these likelihood scores, we compute log-likelihood ratios for the cell type in question relative to the best-fitting cell type across all expected cell types in given query dataset:

$$\:tLLR\left(j\:\right|\:k)=log\left(\frac{{\mu\:}_{k,\:j}}{\underset{{all\:cell\:type\:h}}{{max}}\left({\mu\:}_{h,\:j}\right)}\right)\:$$

For a derivation of how tLLR scores act as a likelihood ratio test, see Supplementary Information.

Secondly, FastReseg would identify the most probable cell type for each individual cell in query dataset based on which reference cell type gives the maximum total $\:tLLR$ score given the cell’s gene expression profile in initial cell segmentation. Then each transcript has an assigned $\:tLLR$ score, $\:tLLR\left(j\:\right|\:k)$ based on its gene *j* and assigned cell type *k*. This score quantifies the alignment of the transcript’s profile with the known characteristics of its most likely cell type *k*, facilitating the initial screening for potential outliers or misclassified transcripts.

### Scoring cells for putative segmentation error

Cells with segmentation errors—especially those located at the boundaries between distinct cell types—are expected to exhibit high spatial dependency in their $\:tLLR$ transcript score profiles. This assumption could be validated by overlaying $\:tLLR$ score profiles on membrane-stained images to confirm discrepancies between segmentation boundaries used for score calculation and the underlying biological structure. FastReseg uses linear regression model to simulate the spatial pattern of $\:tLLR$ score profiles within a given cell and use likelihood ratio test `lrtest()` from `lmtest` R package^14^ to compare it against a null spatial-invariant model to evaluate the degree of spatial dependency:

$$Null~model:~lm\left( {score~\sim 1} \right)$$

$$Spatially~dependent~model:lm\left( {score~\sim ~x+y+z+~{x^2}+~{y^2}+~{z^2}+xy+xz+yz} \right),$$

where *score* is the $\:tLLR$ score of each transcript under the most probable cell type of the query cell and $\:x$, $\:y$, $\:z$ are the spatial coordinates of the transcript. For transcript data with only 2D coordinates, terms associated with $\:z$ are removed from the alternative model.

The spatial dependency score of each cell is then defined as the negative log_10_ value of the p-value from a likelihood ratio test comparing the two models. Cells with scores above a designated threshold are flagged as putative spatial doublets. Of note, FastReseg does not evaluate cells with very few intracellular transcripts due to low statistical power to detect spatial dependency in their transcript scores. The values used in this paper are set to 50 for minimal transcript number allowed in spatial modeling and 3 for minimal spatial dependency score of a cell flagged with putative segmentation error.

### Identification of transcript groups susceptible to segmentation errors

To flag misassigned transcripts, FastReseg first separates transcripts within flagged cells based on the provided cutoff on $\:tLLR$ scores into two classes and then trains a support vector machine (SVM)^11^ to discern the optimal physical boundary that separates well-aligned (score above cutoff) transcripts from poorly aligned (score below cutoff) transcripts in each query cell.

$$svm\left( {class~\sim ~x~+~y~+~z} \right)$$

where $\:z$ term would be removed for 2D transcript data and additional arguments^15^, like $\:kernel$ and $\:gamma$, could be used to control the margin between the two classes of transcripts, adjusting the tightness of decision boundary around the hot spots of the poorly aligned transcripts (Supplementary Fig. S1). A critical tuning parameter is the threshold for calling transcript scores as low versus high: setting this threshold higher produces a much more aggressive flagging of transcripts. FastReseg defaults to a threshold of -2, corresponding to a p-value of 0.046 under the interpretation of transcript score as a log-likelihood ratio between two hypotheses (see Supplementary Information section).

The SVM-based classification approach assumes that misassigned transcripts originate from physically neighboring cells in space and thus transcripts within a physically contiguous low-score region likely belong to the same neighboring cell regardless of their individual $\:tLLR$ scores. Therefore, transcripts predicted by SVM to have low scores are flagged as misassigned transcripts. This approach not only allows for the identification of misassigned cell-type-specific marker transcripts but also extends their impact to neighboring transcripts that may not exhibit strong cell-type-specific expression profiles. In the context of thin tissue sections, one could clean up the current single-cell expression matrix simply by trimming those transcripts, since majority of those misassigned transcripts come from the vertical overlapping of source cells with minor insertions into the assayed tissue section.

Once misassigned transcripts are identified via SVM, FastReseg could further divide them into spatially distinct groups for downstream re-segmentation process if the complete refinement of segmentation is desired. FastReseg offers two methods to segregate those misassigned transcripts in space: density-based spatial clustering (DBSCAN) and Delaunay-based spatial network analysis. The former method uses `dbscan` package^16^ to identify clusters of physically packed transcripts based on their local density at fast speed. The later method leverages `GiottoClass` package^17^ to create a Delaunay network^18^ of those misassigned transcripts and identifies the transcript groups with higher accuracy based on the spatial connectivity between neighboring transcripts, facilitating the detection of potentially separate sources of misassigned molecules. The results used in this paper were generated with density-based method unless noted otherwise.

### Cell segmentation refinement

Each spatially distinct group of misassigned transcripts is considered as potentially originating from different neighboring source cells. To correct those putative segmentation errors, we developed a heuristic-based approach for reassigning groups of transcripts to their correct cellular origins. The process is systematically outlined in the accompanying schematic (Fig. 4), which serves as the foundation for our segmentation refinement methodology. This section details the step-by-step process utilized in our analysis to determine the appropriate refinement action for each transcript group based on their spatial and transcriptomic context.

#### A. Defining heuristic thresholds

Initially, we established specific cutoffs for the number of transcripts and the transcript $\:tLLR$ score for each reference cell type. These thresholds were determined based on the baseline distribution of these metrics across all identified cell types in the query dataset under original cell segmentation. This initial step ensures that subsequent decisions are tailored to the specific characteristics and variability inherent in the dataset for that given cell type.

#### B. Spatial and transcriptomic analysis

The refinement process begins with the identification of each transcript group’s direct neighbors within the tissue’s spatial context. FastReseg constructs a Delaunay network between transcript group and its neighboring cells or groups using their spatial coordinates and defines a direct neighbor based on its minimum molecule-to-molecule distance to transcripts within query group. The distance cutoff used in this paper is 2.7 μm and one can adjust this cutoff based on the observed transcript density, which could be calculated using `FastReseg::runPreprocess()` function. For each group of misassigned transcripts, we assess their $\:tLLR\:$scores under the most probable cell types of itself and its direct neighbors. This dual assessment helps determine the most suitable refinement action based on local transcriptomic profiles.

#### C. Decision-Making for transcript reassignment

The decision tree detailed in our schematic (Fig. 4B) directs the transcript reassignment process, employing a combination of criteria based on transcript number and total $\:tLLR$ score under relevant cell types. Here is a clarified and concise description of this process:

- **New Cell**: A query transcript group is classified as a new cell if it has sufficient number of transcript molecules and a high enough total $\:tLLR$ score under its most probable cell type. This ensures that the new cell is identified based on robust and distinct transcriptomic evidence.
- **Merging**: Transcript groups that do not meet the criteria for a new cell are assessed for potential merging. This assessment considers their highest $\:tLLR\:$score under the most probable cell type of their direct neighbors. A merging event is considered valid if the transcript group aligns transcriptionally with a neighboring cell or group and exhibits a substantial physical merging interface. Specifically, at least 50% of the transcripts within the query group must share the same spatial clusters as those in its adjacent merging candidate. This criterion not only confirms a strong spatial connection between the query cell and its potential merging partner but also maintains the geometric properties of the resulting merged cells. This spatial constraint for merging may be adjusted for tissue samples known to contain elongated cells or cells with protrusions, by modifying the threshold for the minimal fraction of transcripts under shared spatial clusters.
- **Trimming**: Transcript groups that fail to meet the criteria for either a new cell or merging are considered as extracellular. These groups typically contain only a few transcripts and are either transcriptionally distinct from all direct neighbors or lack sufficient spatial clustering overlap with any transcriptionally aligned neighbor. Such groups are likely derived from source cells that were vertically overlapping with the query cell in the original intact tissue but do not retain enough material in the thin tissue section used for the dataset.

These criteria ensure that each transcript group is evaluated based on both quantitative and spatial metrics, providing a systematic approach to accurately reassign those poorly fitted transcript groups in a context-sensitive way in spatial transcriptomics dataset.

### Spatial transcriptomics datasets

The `FastReseg` package (https://github.com/Nanostring-Biostats/FastReseg) contains a small example dataset on FFPE melanoma tissue samples collected on CosMx Spatial Molecular Imager platform. The cell segmentation of this melanoma dataset was conducted with Cellpose models and derived from tissue images with DAPI nuclear stain and immunofluorescence stains against PanCK, CD45, CD298 and DAPI. This dataset al.so includes semi-supervised cell typing results which were generated using `InSituType` package^19^ and a scRNA-seq derived SafeTME matrix published for tumor-immune deconvolution in solid tumors^20^. Six novel clusters were identified in this example dataset and have cluster names as “a” to “f” letters. We use this melanoma dataset to generate figures illustrating the FastReseg workflow. See the manual of package for more information regarding this melanoma example.

The spatial transcriptomics dataset used in Figs. 5 and 6 and Supplementary Fig. S1-S3 to demonstrate the performance of FastReseg algorithm is published by previous study^12^ and could be downloaded from 10.6084/m9.figshare.c.7373860. FFPE sections of clinical biopsy kidney samples were profiled using CosMx Spatial Molecular Imager and the 960-plex CosMx Human Universal Cell Characterization RNA panel. Cell typing and initial cell segmentation was taken as it is from previous study and used to derive reference profiles for FastReseg processing. More than 79 million transcript molecules collected from 123 fields of view (FOVs) covering 76.64 mm^2^ area are included in the kidney dataset and processed by the FastReseg flag-only and full pipelines, both of which ingest the transcriptional data by FOV for small memory footprint and efficient parallel computation. For processing with either Proseg or Baysor transcript-based segmentation methods, the input transcriptional data were split into different files based on their source tissue sections instead.

### Evaluation of marker gene distribution

We use a list of mutually exclusive marker genes of chosen cell types to evaluate the impact of FastReseg’s segmentation refinement step. Cells are grouped by their cell types in the original dataset and included for analysis if they receive trimming of misassigned transcripts. The table below shows the cell-type-specific on-target marker genes used to evaluate the kidney spatial dataset in Figs. 5C and 6D-E.

**Table 1:** Cell-type-specific on-target marker genes used in Figs. 5C and 6D-E.

Cell type	On-target markers
Fibroblast	LUM, COL1A1, COL3A1
Macrophage	CD163, C1QA, C1QC
Plasma (B-cell & Plasmablast)	IGHG2, IGKC, JCHAIN
Podocyte	SPOCK2, PLA2R1, PTGDS, VEGFA
PCT (Proximal.tubule)	DDC, IL17RB, GPX3
Myofibroblast	MYH11, ACTA2

For each cell type, on-target genes are defined as the cell-type-specific marker genes that are characteristic of that particular cell type. Conversely, off-target genes are defined as marker genes that are typically associated with other cell types and thus are the on-target genes of all other cell types in the table above. The “non-marker” category includes all the other genes present in the dataset but not included in the table above. Each subplot in Fig. 5C displays the transcripts number of each gene within cells of the corresponding type in original dataset in x axis and the corresponding transcripts number removed by FastReseg refinement in y axis, providing a clear visual representation of the algorithm’s impact on correcting transcript misassignment in complex tissue samples.

To compare off-target correlation across segmentation methods, we first computed a single-cell metagene score for each cell-type-specific marker group. This score was defined as the sum of transcript counts from all genes associated with the on-target markers of a given cell type group (as listed in Table 1) within each cell. For each tissue section, we then calculated pairwise Pearson correlations between the single-cell metagene score profiles of different marker groups. These inter-group correlations were used as a measure of off-target correlation for each segmentation method within each tissue sample. Figure 6D-E presents the average off-target correlation values between all marker group pairs across all the 10 tissue samples within the CosMx kidney dataset described in Table 3. Each subplot represents a query marker group, showing its correlation with other marker mark groups as stacked bars in distinct filled colors. Segmentation methods are indicated along the x-axis. Figure 6D includes all cells with one or more RNA transcripts in correlation measurement, while Fig. 6E restricts the analysis to cells with 20 ~ 2000 total RNA counts.

### FastReseg processing and computation efficiency characterization

To assess the computation efficiency of FastReseg processing, we subset the CosMx kidney dataset^12^ to contain different number of fields of view (FOVs) with varying number of transcripts and cells in their original cell segmentation, as shown in table below.

**Table 2:** Number of FOV, cells with ≥ 50 counts/cell and Raw transcripts in each subset of CosMx kidney dataset used in Fastreseg processing in Figs. 5D-E and 6.

FOV #	Cell #	Transcript #
5	16,157	4,846,125
10	33,793	10,104,717
20	65,408	19,691,814
50	161,547	44,376,396
100	249,910	68,422,200
123	296,838	79,392,002

Transcriptional data including their gene identity, 3D spatial coordinates and original cell ID assignment come as multiple files in one-file-per-FOV format from the original CosMx assay platform. We found this to be beneficial to keep memory footprint small and speed up the processing since FastReseg workflow does parallel computation at per input file level. Thus, we recommend splitting transcriptional data into image tiles for any spatial datasets collected from other platforms. We then processed those subsets through either the full pipeline or up to the point of isolating misassigned transcripts using `FastReseg:: fastReseg_full_pipeline()` and `FastReseg:: fastReseg_flag_all_errors()` functions, respectively. All the processing included in this study was conducted on an Amazon r5b.4xlarge instance containing 16 vCPU and 128GB memory and set to use 75% of available cores (i.e. 12 cores). The runtime was measured using `time` package in Linux. For peak memory monitoring, we wrapped the FastReseg processing in a single R script, launched it as command line and then used `ps aux` command in Linux to monitor the memory usage of all processes (PIDs) associated with the R script at 10 s interval. The real-time memory usage of FastReseg processes was calculated as the sum of RSS (Resident Set Size, the non-swapped physical memory) across all the relevant processes and the maximum value of the real-time memory usage throughput the pipeline was then reported as the peak memory usage of the entire pipeline in Fig. 5E.

### Processing with Proseg and Baysor

For benchmarking, we processed the same CosMx kidney dataset using 2 additional transcript-based segmentation methods, Proseg (v2.0.4)^21^ and Baysor (v0.7.1)^22^, via the command line provided by the corresponding package. Per recommendations by these 2 algorithms, the input transcriptional data with 3D spatial coordinates in micron units were split into different files based on their source tissue sections and the transcripts from non-RNA probes were excluded from processing by providing the filter pattern on gene names. The original image-based cell segmentation results were fed to the algorithms as prior segmentation results via a transcriptional data column for the original cell ID, whose “0” values indicate extracellular assignment. Proseg processing also receives a column indicating nuclear compartment assignment at transcript level in the original cell segmentation. For Baysor processing, we set 20 as the minimal number of molecules for a cell to be considered as real (`min_molecules_per_cell`), and disabled generation of polygons and associated plots to focus on the primary segmentation alone. The values for all remaining arguments in processing with either algorithm were set to use the default values provided by the corresponding algorithm.

**Table 3:** Number of fovs, cells with positive RNA counts and RNA transcripts in each subset of CosMx kidney dataset used in Proseg and Baysor processing in Figs. 5D-E and 6.

Tissue Sample ID	FOV #	Cell #	RNA Transcript #
1	13	31,568	7,437,310
2	16	23,646	4,923,804
3	12	30,515	9,245,155
4	8	14,970	3,173,182
5	18	61,336	19,085,473
6	8	17,799	4,593,633
7	7	14,063	2,568,850
8	17	39,754	7,077,431
9	11	36,546	6,011,584
10	13	49,772	15,489,886

We conducted all the Proseg and Baysor processing on an Amazon r5b.4xlarge instance containing 16 vCPU and 128GB memory, except for one sample (sample #5) containing 19 million RNA transcripts whose Baysor processing was done on an r5bx12large instance containing 48 vCPU and 384 memory due to its memory consumption exceed the limits allowed by r5b.4xlarge instance. Same as FastReseg characterization, we measured the runtime using Linux `time` package for Proseg and Baysor processing. The time-lapsed memory usage was monitor using Linux `ps aux` command line at 10 s interval and used to calculate the peak memory usage of each process.

## Electronic supplementary material

Below is the link to the electronic supplementary material.

Supplementary Material 1

## Data Availability

The raw datasets analyzed during the current study are published by previous study^12^, Danaher P, et al. *Sci Transl Med.* 2024;16(775):eadl1666 and available at figshare, 10.6084/m9.figshare.c.7373860.

## Code availability

Source code of `FastReseg` package is available at https://github.com/Nanostring-Biostats/FastReseg. The tutorial vignette of the package (https://nanostring-biostats.github.io/FastReseg/articles/tutorial.html) could be used to generate the data for reproducing figures in this study. The processing of example dataset was performed using default configurations unless otherwise noted.

## References

[1] Vasconcelos, A. G., McGuire, D., Simon, N., Danaher, P. & Shojaie, A. Differential Expression Analysis for Spatially Correlated Data, *bioRxiv*, p. 2024.08.02.606405, 1 1 2024. 10.1186/s13059-025-03867-1 PMC12882255 41519776

[2] Vincent, L. & Soille, P. Watersheds in digital spaces: an efficient algorithm based on immersion simulations. *IEEE Trans. Pattern Anal. Mach. Intell.* **13** (6), 583–598 (1991).

[3] Stringer, C., Wang, T., Michaelos, M. & Pachitariu, M. Cellpose: a generalist algorithm for cellular segmentation. *Nat. Methods*. **18** (1), 100–106. 10.1038/s41592-020-01018-x (2021). 10.1038/s41592-020-01018-x 33318659

[4] Greenwald, N. F. et al. Whole-cell segmentation of tissue images with human-level performance using large-scale data annotation and deep learning. *Nature Biotechnology*, **40** (4), 555–565. 10.1038/s41587-021-01094-0 (2022). 10.1038/s41587-021-01094-0 PMC9010346 34795433

[5] Si, Y. et al. FICTURE: Scalable segmentation-free analysis of submicron resolution spatial transcriptomics, *bioRxiv*, p. 11.04.565621, 1 1 2023. (2023). 10.1038/s41592-024-02415-2 PMC11466694 39266749

[6] Park, J. et al. Cell segmentation-free inference of cell types from in situ transcriptomics data. *Nat. Commun.* **12** (1), 6 (2021). 34112806 10.1038/s41467-021-23807-4 PMC8192952

[7] Petukhov, V. et al. Cell segmentation in imaging-based Spatial transcriptomics. *Nat. Biotechnol.* **40** (3), 345–354 (2022). 34650268 10.1038/s41587-021-01044-w

[8] Littman, R. et al. Joint cell segmentation and cell type annotation for spatial transcriptomics, *Molecular Systems Biology*, vol. 17, no. 6, p. e10108, 1 6 (2021). 10.15252/msb.202010108 PMC8166214 34057817

[9] Jones, D. C. et al. Cell Simulation as Cell Segmentation, *bioRxiv*, p. 2024.04.25.591218, 1 1 2024.

[10] 10x Genomics. [Online]. Using Baysor to Perform Xenium Cell Segmentation, 16 02 (2023). Available: https://www.10xgenomics.com/analysis-guides/using-baysor-to-perform-xenium-cell-segmentation. [Accessed 31 10 2024].

[11] Cortes, C. & Vapnik, V. Support-vector networks. *Mach. Learn.* **20** (3), 273–297 (1995).

[12] Danaher, P. et al. Childhood-onset lupus nephritis is characterized by complex interactions between kidney stroma and infiltrating immune cells, *Science Translational Medicine*, vol. 16, no. 775, p. eadl1666, 27 11 (2024). 10.1126/scitranslmed.adl1666 PMC11708815 39602512

[13] Ma, J. et al. X. Yang and Labagn, The multimodality cell segmentation challenge: toward universal solutions, *Nature Methods*, vol. 21, no. 6, pp. 1103–1113, 1 6 2024. 10.1038/s41592-024-02233-6 PMC11210294 38532015

[14] Zeileis, A. & Hothorn, T. Diagnostic checking in regression relationships. *R News*. **2** (3), 7–10 (2002).

[15] Meyer, D., Dimitriadou, E., Hornik, K., Weingessel, A. & Leisch, F. e1071: Misc Functions of the Department of Statistics, Probability Theory Group (Formerly: E1071), TU Wien, 2019. [Online]. Available: https://CRAN.R-project.org/package=e1071

[16] Ester, M., Kriegel, H. P., Sander, J. & Xu, X. A density-based algorithm for discovering clusters in large spatial databases with noise, in *Proceedings of the Second International Conference on Knowledge Discovery and Data Mining*, Portland, Oregon, (1996).

[17] Dries, R. et al. Giotto: a toolbox for integrative analysis and visualization of Spatial expression data. *Genome Biol.* **22** (1), 3 (2021). 33685491 10.1186/s13059-021-02286-2 PMC7938609

[18] Delaunay, B. On the empty sphere, *Bulletin of the Academy of Sciences of the USSR. Class of mathematical and na sciences*, vol. 1934, no. pp. 793–800, 1934. (1934).

[19] Danaher, P. et al. Insitutype: likelihood-based cell typing for single cell spatial transcriptomics, *bioRxiv*, p. 10.19.512902, 1 1 2022. (2022).

[20] Danaher, P. et al. Advances in mixed cell Deconvolution enable quantification of cell types in Spatial transcriptomic data. *Nat. Commun.* **13** (1), 1 (2022). 35046414 10.1038/s41467-022-28020-5 PMC8770643

[21] Jones, D. C. Proseg, 23 04 2025. [Online]. Available: https://github.com/dcjones/proseg.git. [Accessed 25 04 2025].

[22] Petukhov, V. Baysor, 19 11 2024. [Online]. Available: https://github.com/kharchenkolab/Baysor.git. [Accessed 24 04 2025].

