# Supplementary material for: Using transcripts to refine image based cell segmentation with FastReseg
Source: Sci Rep. 2025 Aug 20;15:30508. doi: 10.1038/s41598-025-08733-5 (PMC12368049; doi:10.1038/s41598-025-08733-5)
Supplement: Supplementary file 1 — Supplementary Material 1 [file 41598_2025_8733_MOESM1_ESM.pdf]

Using transcripts to refine image based cell segmentation with FastReseg

Lidan Wu[1], Joseph M. Beechem[1], Patrick Danaher[1]

[1]Bruker Spatial Biology, Seattle, WA, USA 98105

Supplementary Information

Justification of Transcript Score as a Likelihood Ratio Test

Consider a transcript inside a given cell and assume the cell's cell type is known, designated as "cell type 0". We employ a likelihood ratio test to quantify the evidence provided by the transcript to either support or contradict the cell type in question. The null hypothesis posits that the given transcript originates from "cell type 0", while the alternative hypothesis considers the transcript as potentially arising from any other cell type. In gene expression domain, the only data point provided by the transcript molecule is its gene identity. The likelihood ratio between these hypotheses is then computed as follows:

$$LR = \frac{P(gene\ |any\ cell\ type)}{P(gene\ |cell\ type\ 0)} = \frac{\max_{k \in all\ cell\ types}(P(gene\ |cell\ type\ k))}{P(gene\ |cell\ type\ 0)}$$

Likelihood ratio tests operate from the theory that the log-transformed likelihood ratio, $-2log(LR)$, adheres to a chi-square distribution with the appropriate degree of freedom whose value is usually based on the number of parameters in the model. For our setup, we have only one degree of freedom given our choice of cell type that maximizes $P(gene\ |\ cell\ type\ k)$. This implies that a transcript score of -2 (the default threshold of good versus bad fits in FastReseg's SVM step) rejects the null hypothesis with a p-value of 0.046, thereby effectively discriminating transcripts with distinct expression characteristics from the ones in agreement with their host cells' overall expression profiles.

Effects of SVM Configurations on Detection and Refinement of Misassigned Transcripts

To elucidate the impact of different SVM configurations on the processing dynamics of FastReseg across various stages of the workflow, a series of experiments were conducted using a kidney spatial dataset. These explorations, detailed in **Supplementary Figure S1A-D**, investigate the ramifications of altering 3 key SVM parameters: the score cutoff delineating bad-fit from good-fit transcripts, the gamma value of the SVM kernel affecting the model's responsiveness to data complexity, and the cost parameter, which modulates the balance between achieving low error rates and maintaining a smooth decision boundary. The collective influence of these parameters determines the sensitivity and specificity of SVM modeling in identifying regions enriched with poorly fitting transcripts—areas that are potentially indicative of contamination due to segmentation error.

Supplementary Figure S1B reveals a direct correlation between the stringency of the score cutoff and the number of cells with successful detection of misassigned transcripts during the SVM-mediated transcript evaluation phase. Configurations with a more positive transcript score cutoff (Configurations C & D compared to A) tend to identify more cells containing "SVM hits". These hits represent regions where misassigned transcripts are detected via SVM spatial modeling. Consequently, an increase in the number of cells containing SVM hits correlates with a greater total number of flagged misassigned transcripts, as shown in **Supplementary Figure S1D**. Similarly, a higher gamma value (Default versus Configuration A) allows bigger influence of individual below-cutoff transcripts to their spatial neighbors, flagging bigger regions and thus more transcripts as misassigned transcripts. On the other hand, a higher SVM cost value, as seen in Configuration B, imposes stringent penalties on prediction errors, leading to a more complex decision boundary that tightly conforms to the training data. This offsets the effects of a smaller gamma, thereby fine-tuning the SVM's ability to distinguish between good-fit and bad-fit transcripts within the nuanced spatial architecture of the dataset.

Among the parameters tested, the transcript score cutoff exerts the most substantial influence on the outcome of the segmentation refinement process, as evidenced in **Supplementary Figure S1C-D**. More aggressive flagging of transcripts as bad fits, driven by more relaxed score cutoff or elevated gamma values, typically results in a higher proportion of transcripts and cells undergoing complex corrective actions, extending beyond mere trimming to extracellular regions. This aggressive flagging often leads to the emergence of new cell identities from those SVM-predicted bad-fit transcript groups, as they contain enough molecules to provide sufficient cumulative molecular evidence supporting the presence of new cells during refinement. Of note, it's not recommended to use score cutoff much higher than -2, since higher score cutoff means classifying more transcripts with intermediate goodness-of-fit to their current host cells as misassigned transcripts.

These experimental findings illustrate the role of SVM parameter settings in shaping FastReseg's behavior in detecting segmentation errors and further refinement. While the default values used by the FastReseg package provide a good starting point for any new spatial dataset, the user could adjust those parameters (e.g. gamma and cost) to fine tune the sensitivity and specificity of misassignment detection and thus tailor its behavior based on richness of transcriptional information (molecular density in physical space and gene content diversity) and expected cell shape (roundish versus long protrusions) observed in the dataset of interest.

A

Config ID	Score Cutoff	Gamma	Cost
Default	-2	0.4	1
A	-2	0.1	1
B	-2	0.01	500
C	-1.5	0.1	1
D	-1	0.1	1

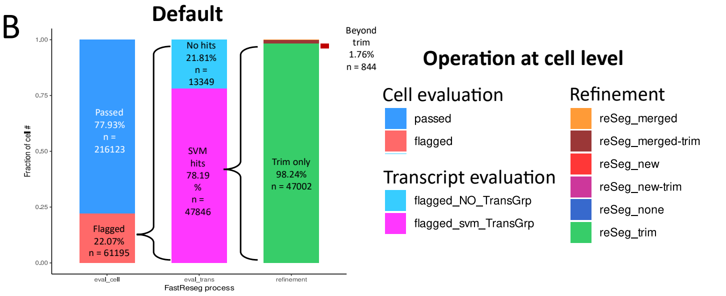

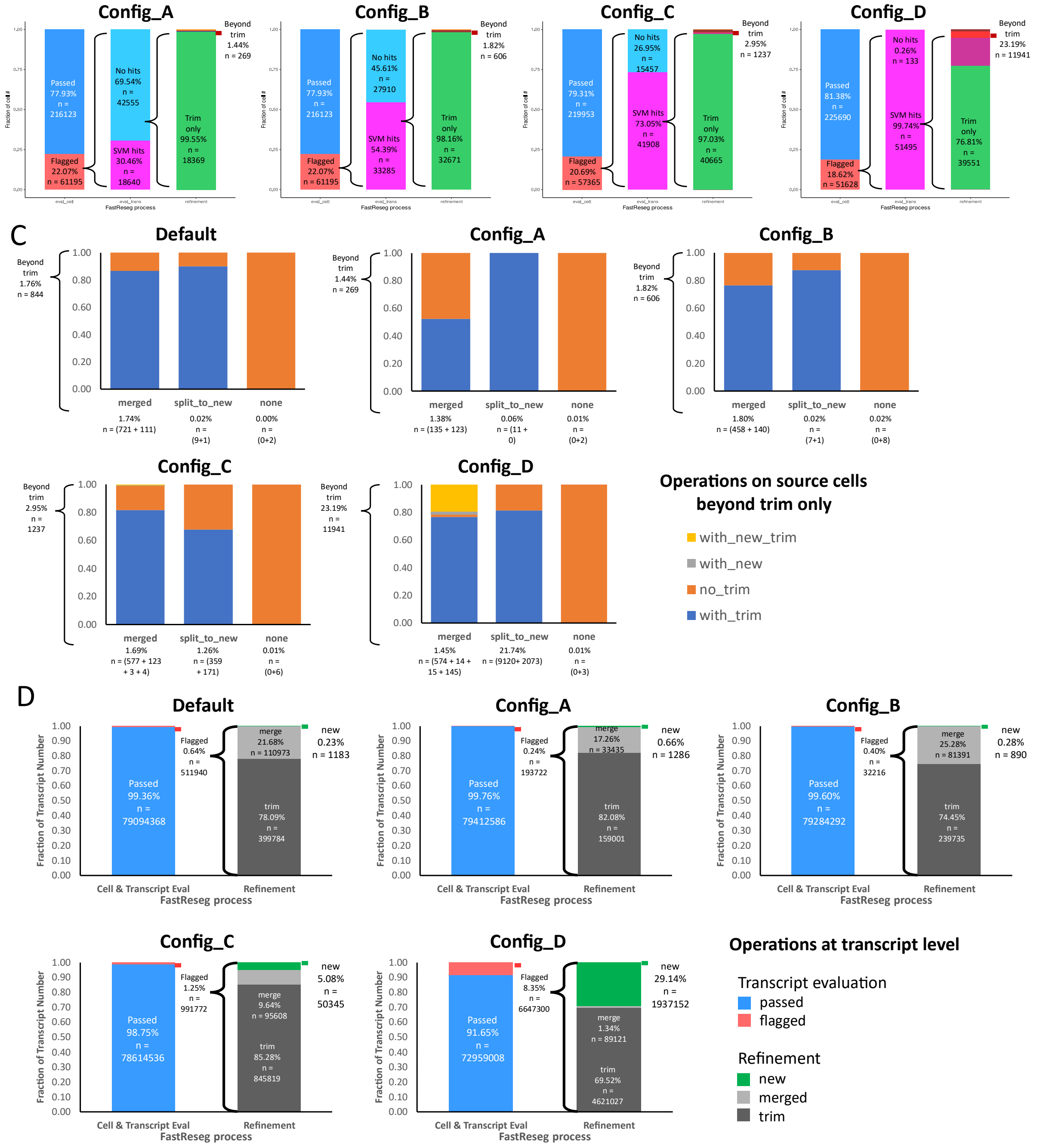

Supplementary Figure S1 Impact of different SVM configurations on FastReseg processing of example kidney dataset. *(A) SVM configurations used for performance comparison in this figure. "Score cutoff" refers to the cutoff on tLLR score to separate*

between well-aligned (score above cutoff) and poorly fit (score below cutoff) transcripts in SVM spatial modeling. Higher score cutoff would classify more transcripts as bellow-cutoff poor fit molecules with respect to the single-cell expression profiles of given cell. "Gamma" and "Cost" are the parameters used by `e1071::svm()` function [1] to define the kernel used in training and prediction, impacting the exact shape of physical boundaries predicted by SVM spatial models. (B) Bar plots on the composition of actions taken at cell level throughout the FastReseg workflow. (C) Bar plots for composition of cell-level segmentation refinement applied to the original host cells beyond simple transcript trimming alone. The number of cells (n) included in each category is noted at the bottom of each bar. Color legend of "with_new_trim", "with_new", "no_trim" and "with_trim" indicates whether the host cells have received additional one or two refinement actions. (D) Bar plots on the breakdown of operations conducted at the transcript level.

Illustrative Examples of FastReseg Processing Journey

To provide a better understanding of FastReseg's workflow, we offer a visual narrative showcasing the distinct outcomes of its full pipeline, illustrated through examples of cells that underwent various actions during the processing of the CosMx kidney dataset. **Supplementary Figure S2** delineates different refinement scenarios into separate panels. In Panel A, we observe cells whose flagged transcripts have been allocated to form new cells. Panel C follows cells with transcripts that were merged into adjacent cells, while Panel D focuses on cells whose transcripts were trimmed to extracellular space. Each panel illustrates the comprehensive journey of cell transcripts across rows, from initial inputs to the final refinement outcomes. Additionally, **Supplementary Figure S2B** shows the single-cell distribution of spatial dependency score from the kidney dataset's spatial doublet test results. Notably, cells that split into new cells (red line) exhibit strong spatial dependency in their assigned transcript scores. Meanwhile, cells involved in merging events (orange line) display a binomial distribution in their spatial dependency score, with a subpopulation corresponding to cells receiving transcripts from neighboring cells and showing score below the flagging cutoff (dashed black line, cutoff = 3).

The inputs of FastReseg process are depicted in rows (i) to (iii). Row (i) showcases the morphological stains that provide the visual basis for initial image-based segmentation. Row (ii) features the spatial distribution of canonical marker genes and other transcripts, highlighting the cellular composition prior to refinement. Row (iii) reveals the cell type assignments provided by the original dataset given the initial segmentation borders, setting the stage for further evaluation. Notably, cells with faint CD45 staining (red color in Row i) often have transcripts for immune-specific marker genes (Row ii). However, they are not always identified as immune cells in the cell typing results (Row iii) due to the presence of non-immune-specific marker genes. This suggests that immune cells might be present in the vertical z direction, but their cell volume may have been mostly excluded from the dataset during tissue sectioning.

The first tier of FastReseg process, presented in Row (iv) and (v), details the spatial doublet test designed to identify cells with putative segmentation errors. Row (iv) shows the spatial pattern of the transcript scores under each cell's most probable cell type, while Row (v) reveals the spatial dependency values for each cell. Markedly, cells containing mutually exclusive marker genes and cells with boundary error between different cell types have higher spatial dependency scores and are flagged by the spatial doublet test.

The second tier of FastReseg process involves identification and segregation of the misassigned transcripts within the flagged cells. In Row (vi), the decision boundary between good and bad fits within each flagged cell is visualized by coloring each transcript according to the decision values predicted by the corresponding SVM model. Row (vii) then colors the transcripts based on their group assignments, with non-flagged transcripts shown in gray and spatially segregated misassigned transcripts in bright

colors. Transcripts flagged with the same color are considered to originate from the same source cell and are evaluated as a group in the downstream refinement stage.

The culmination of FastReseg's refinement process is captured in Row (viii) and (ix), where transcripts are depicted as dots colored according to their cell assignment before (Row ix) and after (Row ix) refinement. The 2D footprints of original cell segmentation results are represented as dim shadows beneath these transcript dots. Bright circles highlight regions where the cellular assignment of flagged transcripts has changed, demonstrating the impact of the FastReseg refinement.

Altogether, this figure serves as a comprehensive visual summary of FastReseg process, from initial segmentation and identification of potential errors to the refinement and reassignment of transcripts. Each step, visualized through detailed rows, highlights the method's ability to detect and correct segmentation inaccuracies through a modular and systematic approach.

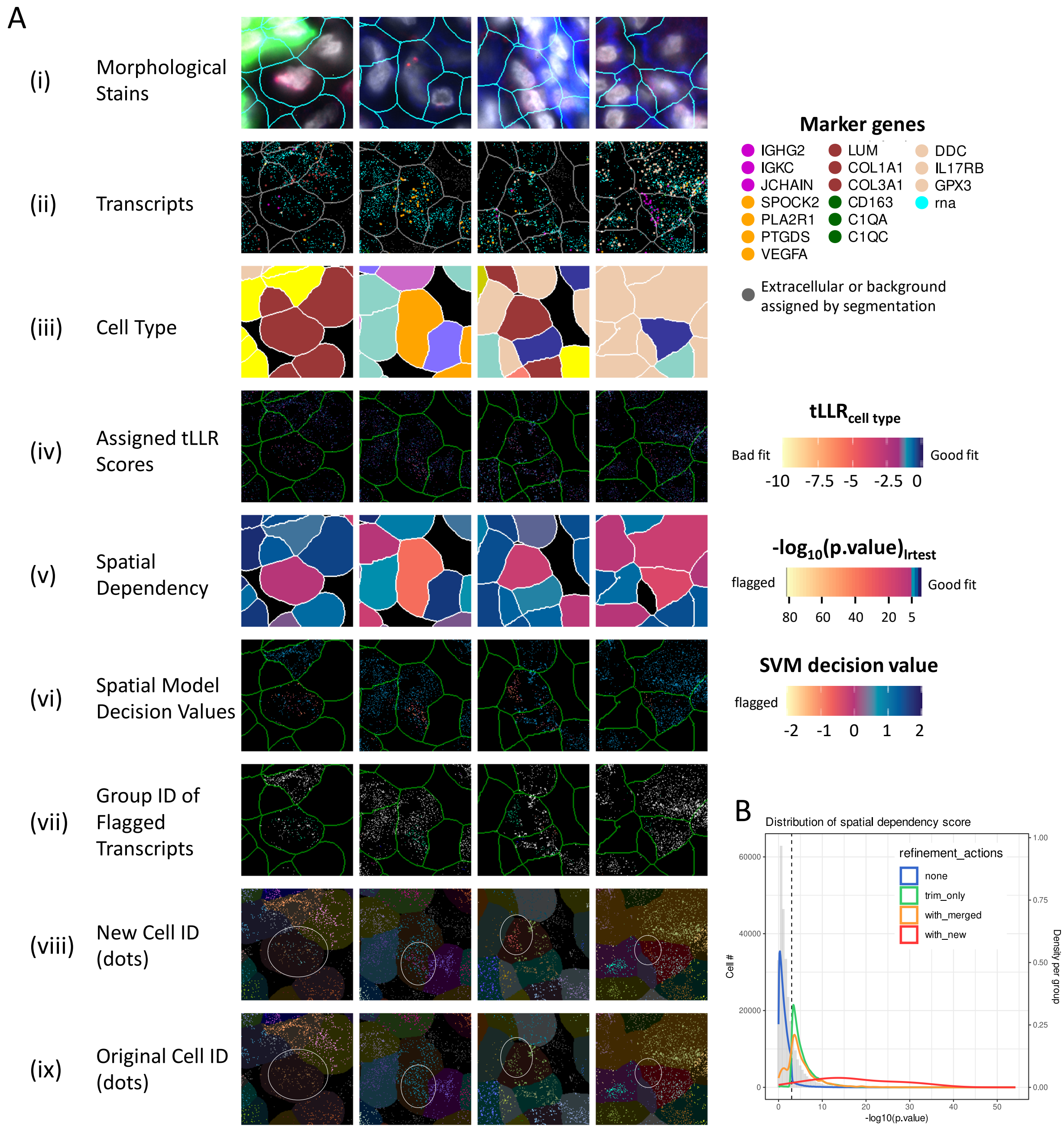
A
(i) Morphological Stains
(ii) Transcripts
(iii) Cell Type
(iv) Assigned tLLR Scores
(v) Spatial Dependency
(vi) Spatial Model Decision Values
(vii) Group ID of Flagged Transcripts
(viii) New Cell ID (dots)
(ix) Original Cell ID (dots)
Marker genes
IGHG2
IGKC
JCHAIN
SPOCK2
PLA2R1
PTGDS
VEGFA
LUM
COL1A1
COL3A1
CD163
C1QA
C1QC
DDC
IL17RB
GPX3
rna
Extracellular or background assigned by segmentation
tLLRcell type
Bad fit
Good fit
-10 -7.5 -5 -2.5 0
-log10(p.value)lrtest
flagged
Good fit
80 60 40 20 5
SVM decision value
flagged
-2 -1 0 1 2
B
Distribution of spatial dependency score
refinement_actions
none
trim_only
with_merged
with_new
Cell #
Density per group
-log10(p.value)

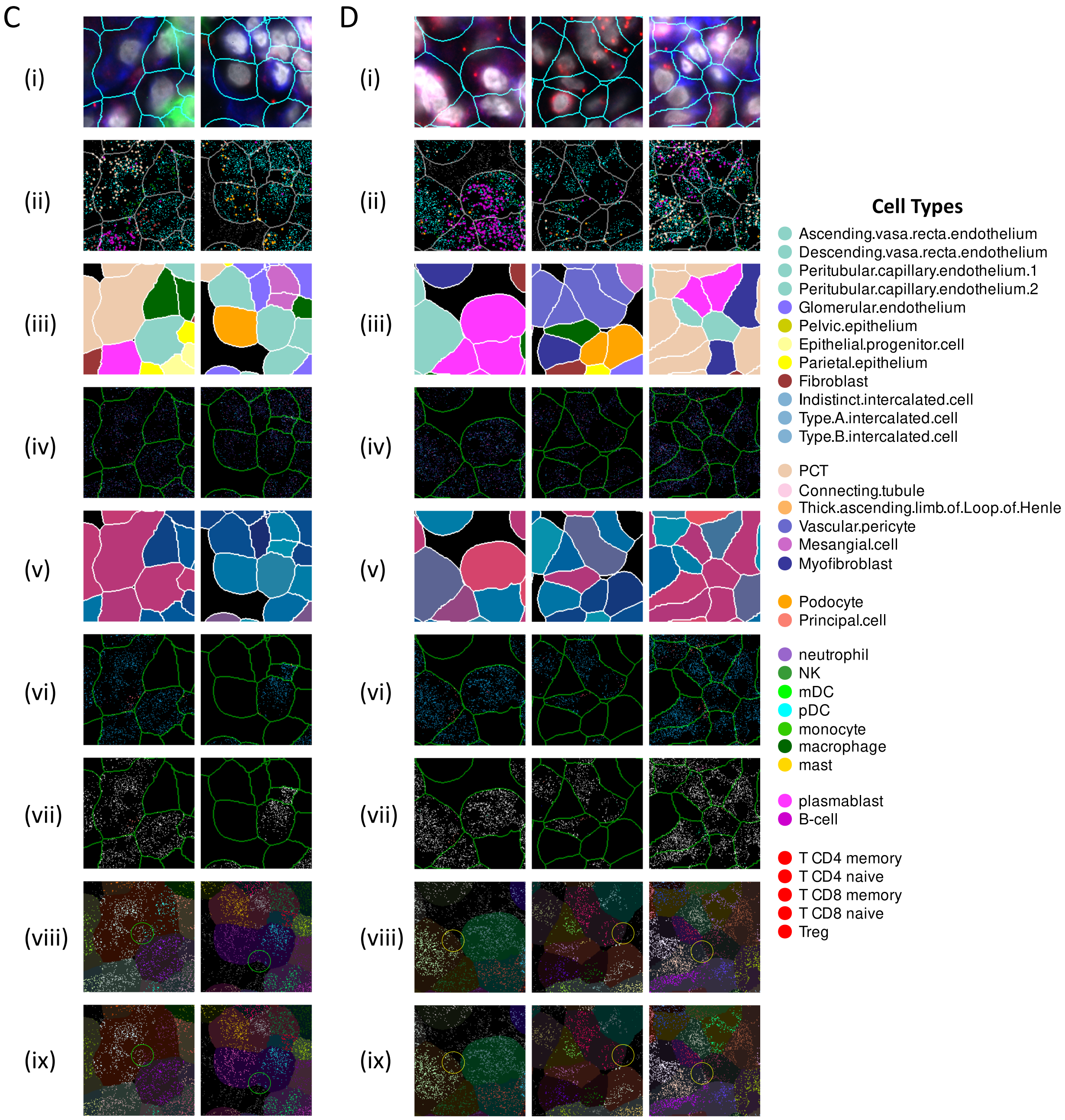

Supplementary Figure S2 Illustrative Examples of FastReseg Process*. Cells that underwent different resegment actions in FastReseg's full pipeline processing of CosMx kidney dataset were used as demostrative examples. (A, C-D) Panels of 2D spaital plots illustraing various metrics for selective query cells and their 300 px X 300 px (54 µm) neighborhood. Shared color legends across Panel A, C and D indicates cells forming new cells, merging, of having misassigned transcripts removed. Row (i-ix) depict sequential steps of the FastReseg process. Inputs for FastReseg include (i) initial cell segmentation (cyan) derived from morphological stains (PanCK: green, CD298: blue, DAPI: gray), (ii) transcripts' spaital coordinates, colored by cell types for canonical markers and cyan dots for non-marker genes, (iii) original dataset's cell typing results based on initial segmentation (cell areas colored by cell types). The cell-level error detection is shown in (iv) spatial distribution of assigned transcript tLLR scores under each cell's most probable cell type, and (v) spatial dependency score from the spaital doublet test. The transcript-level error detection is visualized with (vi) the decision value from SVM spatial modeling to classify good vs. bad fits, and (vii) the group ID assignment given physcial segregation of flagged misassigned transcripts (white for non-flagged transcripts, bright colors for flagged groups). To visualize the refinment actions' impacts, transcritps are colored based on their cellular assignment post-FastReseg refinement (viii) and in their original dattaset (ix) for comparison. For visual tracking, original cell segmenation's 2D footprints are shown as dim shadows underneath transcript dots. Regions with changed cellular assingment of flagged*

transcripts are highlighted by bright circles, emphasizing context-dependent refinement. Panel (B) shows histogram (gray bars, left y-axis) and density curves (colorful lines, right y-axis) of single-cell spatial dependency scores observed in the CosMx kidney dataset during the spatial doublet test. Cells are grouped by their final refinement actions. The cell-level error flagging cutoff is marked as a black vertical dashed line at $-log_{10}(p.value) = 3$.

Behavioral Differences Across Transcript-Informed Segmentation Methods

Supplementary Figure S3 presents a qualitative, head-to-head comparison of transcript-aware segmentation methods applied to identical regions of interest (ROIs) from the CosMx kidney dataset. While not intended as a comprehensive performance benchmark, this figure highlights characteristic behavioral differences among methods in assigning transcripts to cells. FastReseg (Column 4) selectively reassigns transcripts in cases where transcriptional evidence supports deviation from the original image-based segmentation (Column 3), while otherwise preserving the original morphological boundaries. The transcriptional evidence used by FastReseg to guide these adjustments, such as localized clusters of transcripts with low assigned $tLLR$ scores, can be visualized in Row ix of **Supplementary Figure S2**. In contrast, other transcript-based methods introduce more extensive alterations. ProSeg (Column 5) frequently modifies cell borders and generally maintains continuity across z-planes. However, it exhibits two notable error modes: in Row 4, ProSeg overextends a cell's 2D footprint into a neighboring region lacking morphological signal (Column 1), producing an abnormally large cell with inflated transcript count and area; in Row 5, it erroneously merges two adjacent cells into a single segment, despite clear morphological separation, leading to loss of cell identity resolution. Baysor (Column 6), on the other hand, suffers from over-splitting errors, likely influenced by the 0.8 µm spacing between transcript coordinates across imaging planes, which results in overlapping and mixed cell identities when projected onto 2D. These qualitative examples illustrate how FastReseg achieves biologically grounded refinements without over-segmentation or boundary distortion, striking a balance between transcriptional evidence and morphological context.

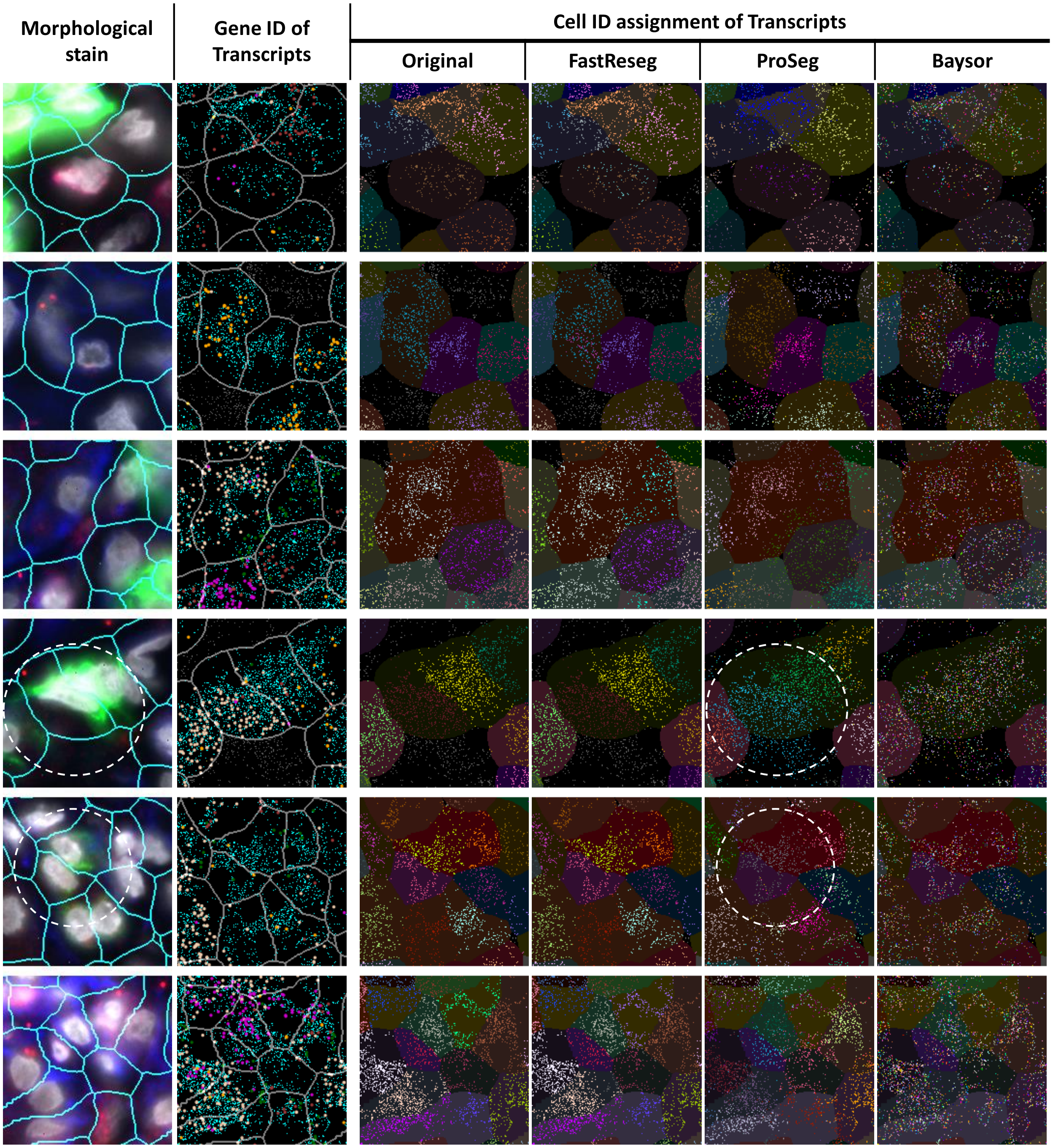

***Supplementary Figure S3 Example ROI Comparision of Transcript-Aware Segmentation Methods**. A head-to-head visual comparision of different segmentation methods applied to the same region of interest (ROI) from the CosMx kidney dataset. The layout follows a column-wise method arrangement and a row-wise organization of ROIs, with each panel displaying 2D spatial plots using the same color legends as in **Supplementary Figure S2**. Column 1 shows the intial image-based cell segmentation borders (cyan) oerlaid on the morphological stains (PanCK: green, CD298: blue, DAPI: gray). Column 2 displays the spatial distribution of transcripts, with dots colored by canonical marker gene identity and non-marker genes shown in cyan. Column 3-6 depict transcript-to-cell assignments derived from different cell segmentation methods: original image-based cell segmentation (Column 3), FastReseg (Column 4), ProSeg (Column 5), and Baysor (Column 6). Transcripts assigned to extracellular space—either marked as background or noise by ProSeg or Baysor, or having a confidence score below 0.9 in Baysor—are colored dark gray. To avid visual tracking, the original cell segmenation's 2D footprints are shown as dim shadows beneath the transcript dots. The circled region in Row 4 highlights a common error mode observed in ProSeg, where the 2D footprint of a cell was*

expanded well beyond the morphologically defined cell boundaries into a neighboring area lacking morphological evidence of a cell. This resulted in abnormally large segmented cells with inflated 2D area, volumne and transcript count. In Row 5, another ProSeg error mode is shown in circled region, where two adjacent cells were incorrectly merged into a single unit despite clear separation in the underlying morphology, resulting in loss of cell boundary fidelity. These examples illustrate distinct behavioral patterns of transcript-aware segmentation methods; this figure is intended as a qualitative comparison and not a comprehensive performance benchmark.
